# Synthesis and binding studies of two new coumarin-squaramide-based receptors for NSAIDs

**DOI:** 10.1039/d5ra08698a

**Published:** 2026-01-19

**Authors:** Luca Mancini, Filippo Ingargiola, Giampaolo Barone, Patrizia Rossi, Mauro Formica, Eleonora Macedi, Martina Lippi, Luca Giorgi, Luca Prodi, Vieri Fusi, Daniele Paderni

**Affiliations:** a Department of Pure and Applied Sciences, University of Urbino Via Ca’ Le Suore 2-4 Urbino 61029 Italy luca.giorgi@uniurb.it vieri.fusi@uniurb.it; b Department of Chemistry “Giacomo Ciamician”, Università degli Studi di Bologna Via Selmi 2 40126 Bologna Italy luca.prodi@unibo.it; c Department of Biological, Chemical and Pharmaceutical Sciences and Technologies (STeBiCeF), University of Palermo Viale delle Scienze, Edificio 17 90128 Palermo Italy; d Department of Industrial Engineering, University of Florence Via S. Marta 3 Florence 5013 Italy

## Abstract

Two new squaramide-based receptors containing coumarin units have been synthesized and characterized in both solution and solid states. L1 (3-(benzylamino)-4-((2-oxo-4-(trifluoromethyl)-2*H*-chromen-7-yl)amino)cyclobut-3-ene-1,2-dione) is a linear molecule, while L2 (4,4′-((1,3-phenylenebis(methylene))bis(azanediyl))bis(3-((2-oxo-4-(trifluoromethyl)-2*H*-chromen-7-yl)amino)cyclobut-3-ene-1,2-dione)) is an open chain ligand which results in the ditopic form of the simpler parent ligand L1. The new molecules have been designed to act as receptors for non-steroidal anti-inflammatory drugs (NSAIDs) possessing both squaramide units as double hydrogen bond (HB) donor sites that are able to interact with the carboxylate functions of the guests, and 4-trifluoromethylcoumarin moieties as aromatic photoactive domains to facilitate π-stacking or hydrophobic interactions with the drug's aromatic rings. The ability of the new receptors to interact with benzoate (BzO^−^), ibuprofen (IBU^−^), naproxen (NPX^−^) and ketoprofen (KET^−^) sodium salts was studied *via* UV-Vis and fluorescence spectroscopy, ^1^H-NMR measurements and DFT calculations. Finally, mass spectrometry studies demonstrated that L1 showed the tendency to form adducts with a 2 : 1 ligand-to-anion stoichiometry ([L1_2_-Anion]^−^), while only adducts with a 1 : 1 stoichiometry ([L2-Anion]^−^) were visible for L2.

## Abstract 2

New squaramide-based receptors for NSAIDs bearing coumarin units have been synthesized and characterized in solution and solid state, revealing able to interact with the guest anions *via* H-bonding between squaramide and carboxylate functions.

## Graphical abstract

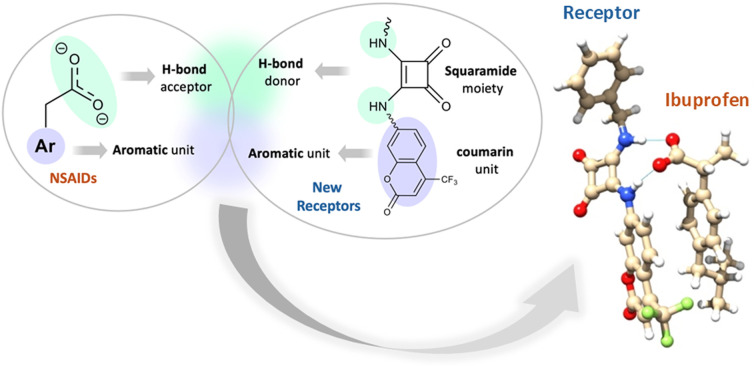

## Introduction

Since the early days of supramolecular chemistry, one of the most popular research areas is the development of receptors for anions.^1–7^ To this purpose, many efforts have been made to understand the appropriate structural requirements to achieve ligands that are able to selectively interact with specific anions. In this context, the focus has mainly centered on exploiting the increasing knowledge of anion coordination chemistry, obtaining systems that can signal interaction with anionic guests by changing an intrinsic chemical-physical property.^8–10^ In the last decades, following the different research needs, much attention has been given to the design and synthesis of chemosensors for emerging pollutants (EPs). Due to the endless release of anthropogenic chemicals into the environment, there is a wide array of compounds with potential ecological and toxicological risks that are still not fully understood. Nowadays, one of the most impactful classes of EPs is represented by active pharmaceutical ingredients (APIs) and their metabolites. The most critical point of pharmaceuticals as EPs lies in the chemical structural characteristics by which drugs are designed, namely the highly stable chemical structure and the presence of biologically active functional groups.^11^ These small molecules, typically ranging from 200 to 500/1000 molecular weight, often feature acidic or/and basic functionalities. Consequently, depending on the environmental conditions, they may be in their cationic, anionic, neutral or zwitterionic form.^12^

**Fig. 1:**
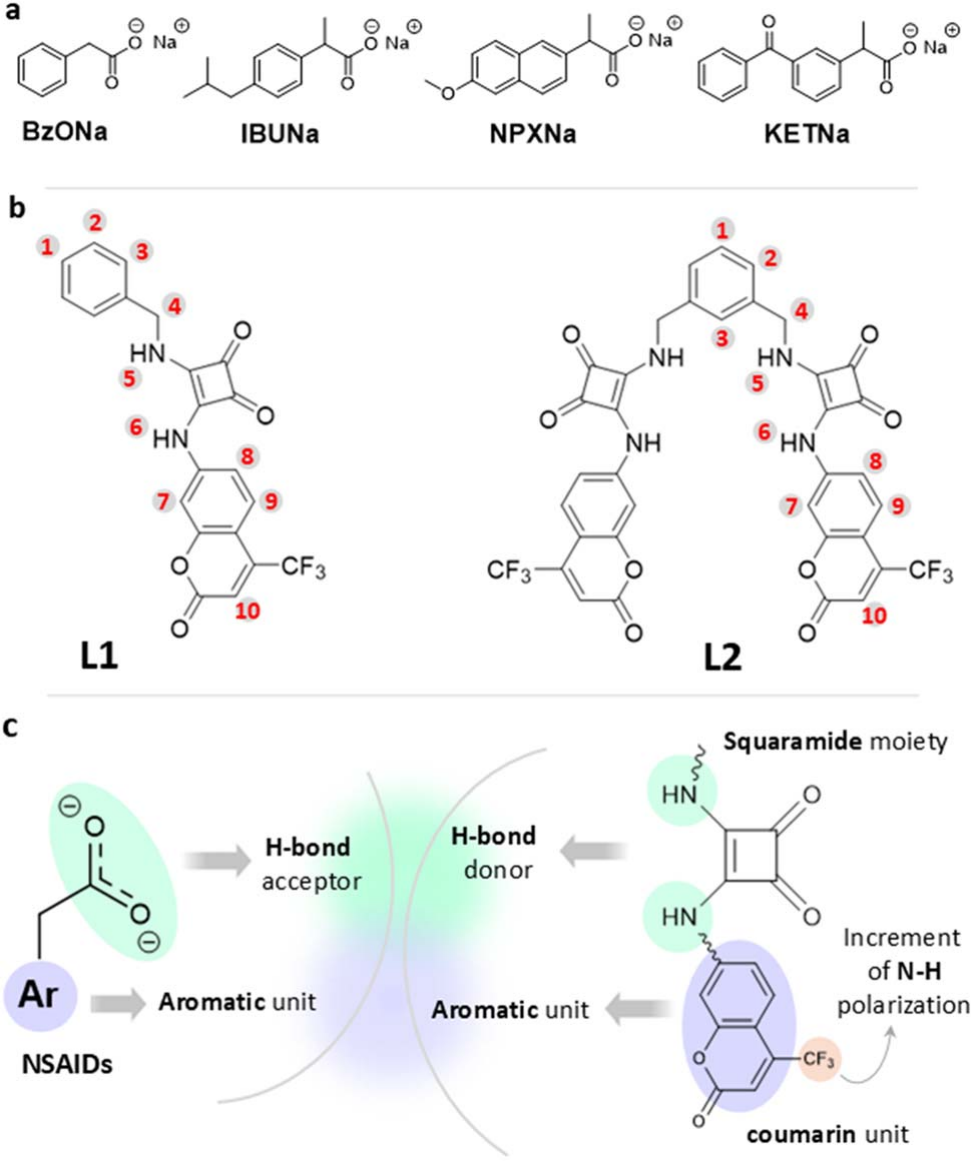
(a) Molecular structures of the NSAIDs and benzoate sodium salts studied in this paper; (b) structures of ligands L1 and L2 together with ^1^H NMR labelling; and (c) schematic for the ligand design.

Among all, non-steroidal anti-inflammatory drugs (NSAIDs) provide one of the most frequently detected EPs in aquatic environment such as waste waters, effluents and surface waters.^13,14^ Their widespread consumption is due to their analgesic, antipyretic and anti-inflammatory effects caused by the inhibition of cyclooxygenase (COX) enzymes, which catalyse the synthesis of several prostaglandins from arachidonic acid.^15^ In the early 2000s, it was estimated that about 30 million people made use of NSAIDs daily,^16^ representing 5–10% of global prescriptions.^17^ Among the traditional NSAIDs, the most widely used are ibuprofen (IBU), naproxen (NPX) and ketoprofen (KET) (Fig. 1a). Indeed, several studies demonstrated their presence in European and American river water samples, emphasizing the need to understand the impact and consequences that these new contaminants could have on both human health and the enviroment.^18,19^ A recent work that combined data of the concentration values obtained for analgesics/anti-inflammatories drugs, pertaining to hundreds of municipal wastewater treatment plants from various global locations, showed a concentration range between ng L^−1^ to µg L^−1^.^20^ Most NSAIDs present a carboxylic acid moiety and they are mostly administered as salts. Being weak acids (p*K*_a_ values between 3.5–4.5),^21^ they are mostly present in environmental aqueous matrices in their anionic forms.^22,23^

At present, the methodologies used for detecting EPs in water rely on high-performance liquid chromatography (HPLC) and gas chromatography (GC) coupled with various detection systems, capillary electrophoresis, and mass spectrometry analysis.^24–27^ All the mentioned techniques require preliminary time-consuming derivatization procedures and the optimization of a large number of parameters, such as temperature, sample collection, cost, time, specialized operators, *etc.*

These limitations necessitate the research of chemical sensors as suitable alternatives that are capable of the rapid and on-site preliminary detection of EPs, which would allow for increasing environmental monitoring while reducing operational costs.^28–30^ Over time, several design strategies have been implemented to obtain molecular systems that are able to interact with carboxylate anions. Metal complexes with an unsaturated metal core^31,32^ and neutral organic molecules containing hydrogen bond donor groups^33,34^ represent the most common systems that can be found in the literature. In the latter case, urea- and more recently squaramide (3,4-diamino-3-cyclobuten-1,2-dione)-based compounds have been largely developed due to the acidity of the –NH functional groups, which provide the host molecules with directional binding sites, making them exceptional double hydrogen bond (HB) donors towards carboxylates.^35,36^ Several works have proven that squaramide functional groups are better HB donors than urea even in highly competitive media, a feature ascribable to the increment of the aromaticity of the squaramide ring upon anion complexation.^37^ Despite these possible advancements, squaramide-based receptors have not yet been sufficiently explored for NSAIDs recognition, since just a few squaramide-based ligands designed for this purpose have been recently reported.^38,39^

With the aim to further broaden the case studies, in the present work we report the design, synthesis and characterization of two new squaramide-based ligands (Fig. 1b): the linear molecule L1 (3-(benzylamino)-4-((2-oxo-4-(trifluoromethyl)-2*H*-chromen-7-yl)amino)cyclobut-3-ene-1,2-dione) and the open chain ligand L2 (4,4′-((1,3-phenylenebis(methylene))bis(azanediyl))bis(3-((2-oxo-4-(trifluoromethyl)-2*H*-chromen-7-yl)amino)cyclobut-3-ene-1,2-dione)), with L2 being the ditopic form of L1. Since NSAIDs are normally composed of a carboxylate group linked to an aromatic portion, we designed these receptors by inserting squaramide groups as the HB donor sites that are able to interact with the carboxylates, and 4-trifluoromethylcoumarin moieties as aromatic photoactive domains that are able to facilitate π-stacking or hydrophobic interactions with the drug's aromatic rings (Fig. 1c). The presence of a trifluoromethyl moiety, being a powerful electron-withdrawing group, should increase the N–H polarization and might help with avoiding self-association, thus preventing aggregation phenomena.^40,41^ The ability of L1 and L2 to interact with NSAIDs such as IBU^−^, NPX^−^, KET^−^, and sodium benzoate (BzO^−^), selected to test the response of the ligands to a simpler aromatic carboxylate, was studied *via* UV-Vis and fluorescence spectroscopy, ^1^H-NMR measurements, mass spectrometry and DFT calculations.

## Results and discussion

### Synthetic procedures

**Scheme 1:**
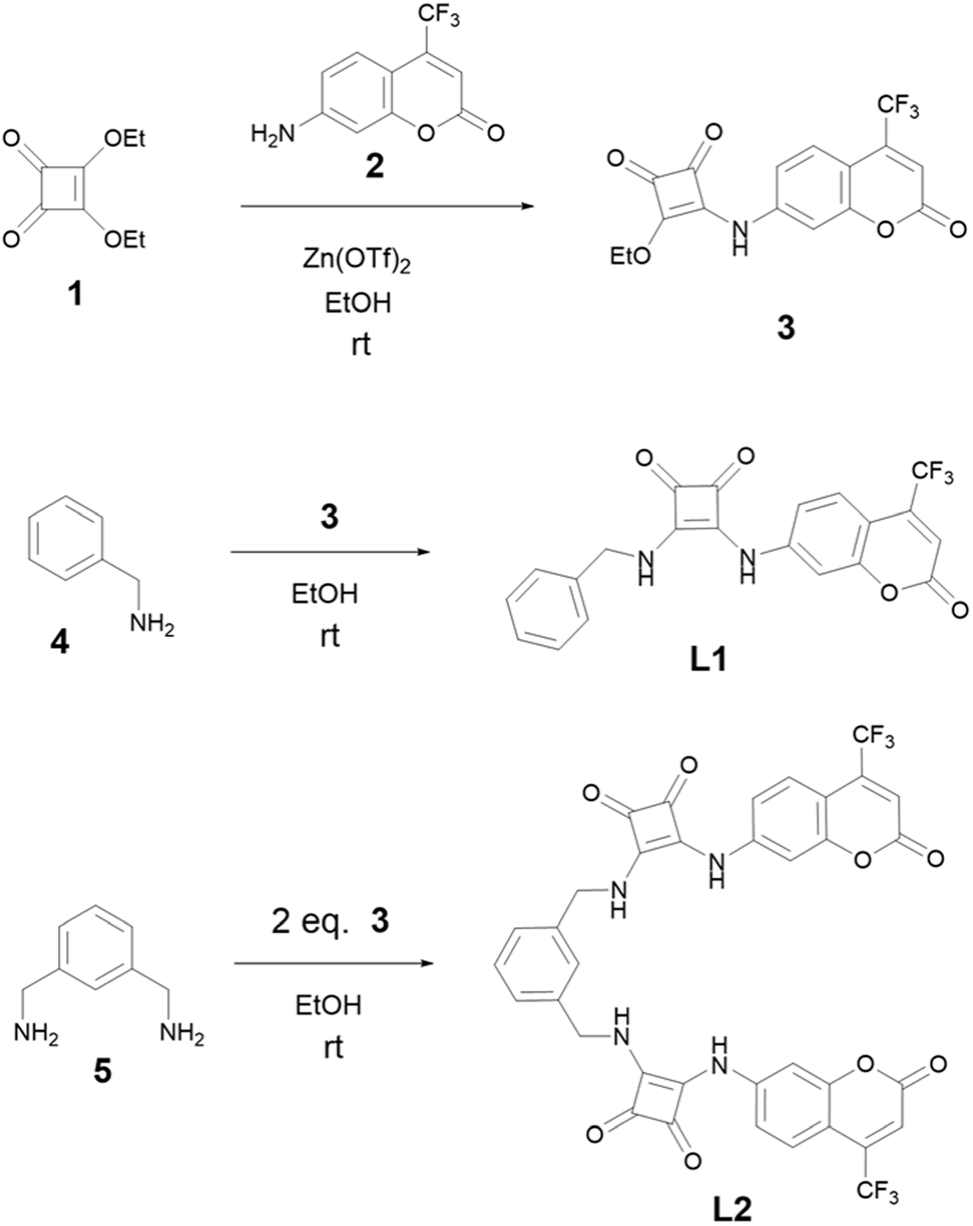
Synthesis pathways of L1 and L2.

The strategy for the synthesis of L1 and L2 is reported in Scheme 1. Intermediate 3 was synthesized by reacting commercially available 7-amino-4-(trifluoromethyl)coumarin (2) and diethylsquarate (1) obtained as previously reported,^42^ using 20% mol of zinc triflate as the catalyst. Squaramide ligands L1 and L2 were obtained by coupling one equivalent of the intermediate 3 with one equivalent of the amine 4 (benzylamine) for L1, and by mixing two equivalents of 3 with 1,3-bis(aminomethyl)benzene (5) in the case of L2. The reactions were conducted in ethanol at room temperature without any catalyst. The final products precipitated directly from the reaction mixture and were purified in good yields by crystallization from hot ethanol.

### X-ray crystal structure of L1

**Fig. 2:**
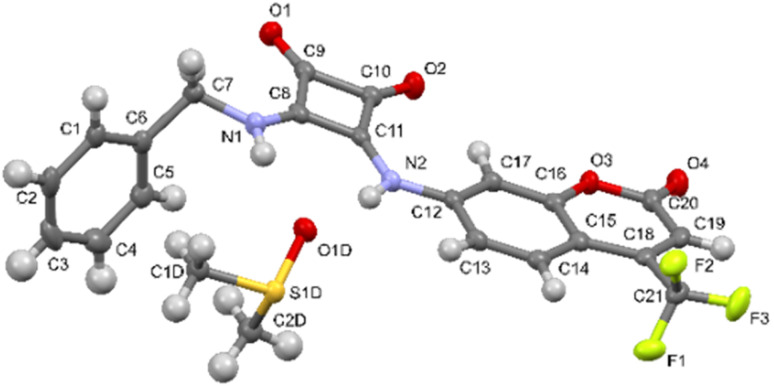
View of the asymmetric unit of L1·DMSO; Ortep3 view with 50% ellipsoid probability is shown. For the sake of clarity, only the label scheme of the non-hydrogen atoms is reported.

In the asymmetric unit of the L1·DMSO crystal structure, one molecule of ligand L1 and one molecule of DMSO are present (see Fig. 2).

**Scheme 2:**
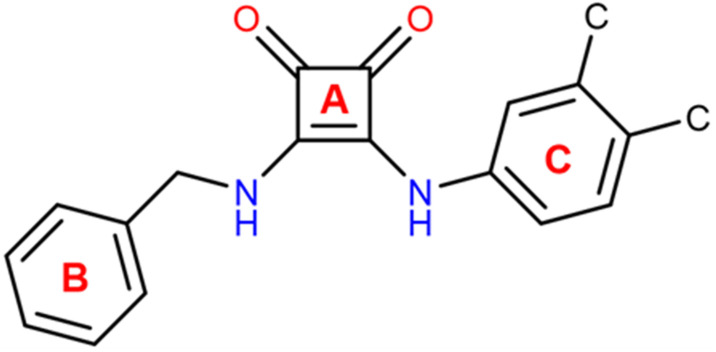
Fragment searched in the CSD. The letters A, B and C refer to the mean planes containing the non-hydrogen atoms of the three rings used to determine the angles between the squaramide ring and the two aromatic moieties.

In ligand L1, the squaramide ring and the coumarin moiety are nearly coplanar, forming an interplanar angle of 8.67(4)° as defined by their non-hydrogen atoms. The phenyl ring forms an angle of 75.30(7)° with the plane of the squaramide unit, while the angle between the planes defined by coumarin and the phenyl fragment is 83.95(7)°. These values are consistent with those reported in the Cambridge Structural Database (CSD, online version)^43^ for compounds containing the structural fragment shown in Scheme 2 [angle A–B: 54.8°–81.0°, mean 66.8°; angle A–C: 3.1°–25.9°, mean 15.6°; angle B–C: 50.4°–80.2°, mean 66.8°].

**Fig. 3:**
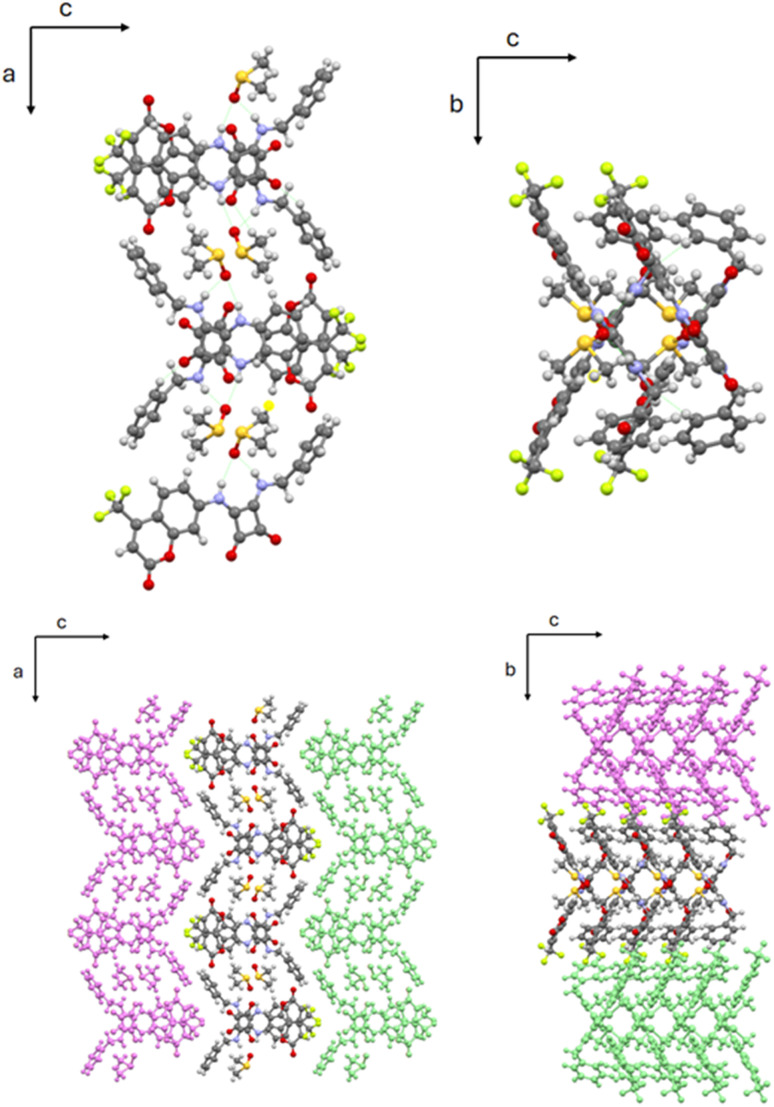
Top, left: view of the L1·DMSO chain along the *b* axis; right: view of the L1·DMSO chain along the *a* axis. Bottom, left: view of the crystal packing of L1·DMSO along the *b* axis; right: view of the crystal packing of L1·DMSO along the *a* axis.

In the L1·DMSO complex, the ligand and solvent molecules are linked *via* two strong N–H_L_⋯O_DMSO_ hydrogen bonds and two weaker C–H⋯O interactions (see Table 1). These hydrogen bonding interactions lead to the formation of a one-dimensional chain extending along the *a*-axis (see Fig. 3).

**Table 1:** Selected intermolecular interactions in L1·DMSO

X–H⋯Y	X⋯Y (Å)	H⋯Y (Å)	X–H⋯Y (°)
N1–H1N⋯O1D	2.895(3)	2.05(3)	161(3)
N2–H2N⋯O1D	2.794(2)	1.95(3)	167(3)
C5–H5⋯O1^a^	3.475(3)	2.60(3)	153(2)
C2D-H2DB⋯O2^a^	3.342(3)	2.40(3)	158(3)

a = −*x* + 1.5, +*y* − 0.5, +*z*.

**Fig. 4:**
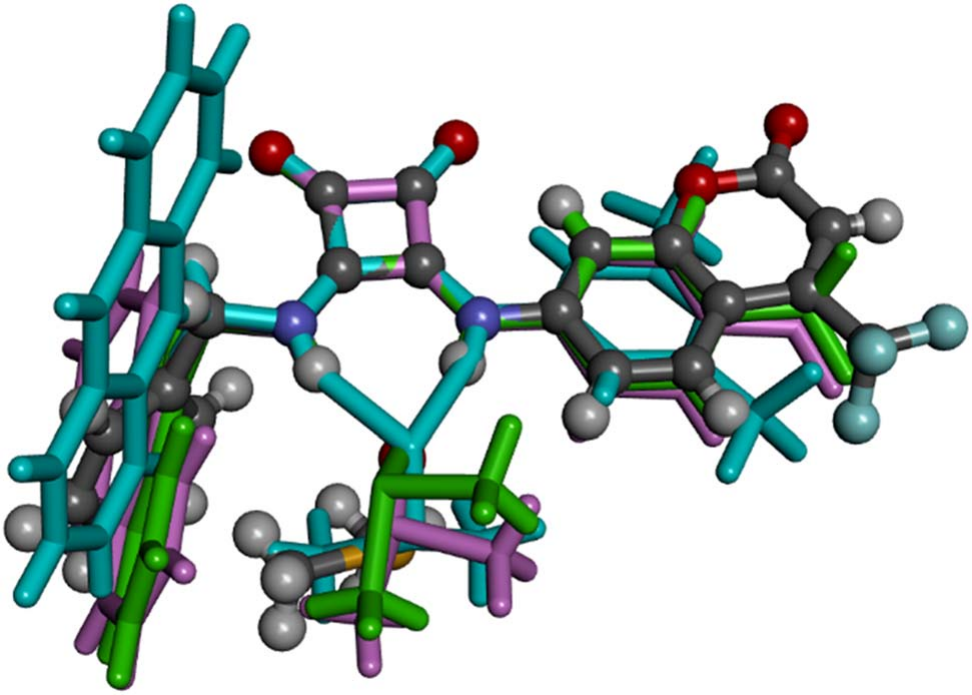
Superimposition of L1·DMSO (ball and stick) with CIPYIL (stick, pale blue), CIPYOR (stick, pale green) and CIPYUX (stick, pale pink).

Among the five structures retrieved from the CSD that feature the fragment depicted in Scheme 2, three crystallize with one DMSO molecule per asymmetric unit. In these three structures (CSD ref. codes: CIPYIL, CIPYOR, and CIPYUX),^44^ the ligands incorporate an anthracene moiety in place of the phenyl ring found in ligand L1, and a *para*- or *meta*-substituted phenyl derivative instead of the coumarin moiety (see Scheme S1). Excluding CIPYIL, the remaining two structures are closely comparable to L1·DMSO (see Fig. 4), and all three exhibit chelation of the DMSO oxygen atom by the squaramide nitrogen atoms.

### Photophysical characterization

**Fig. 5:**
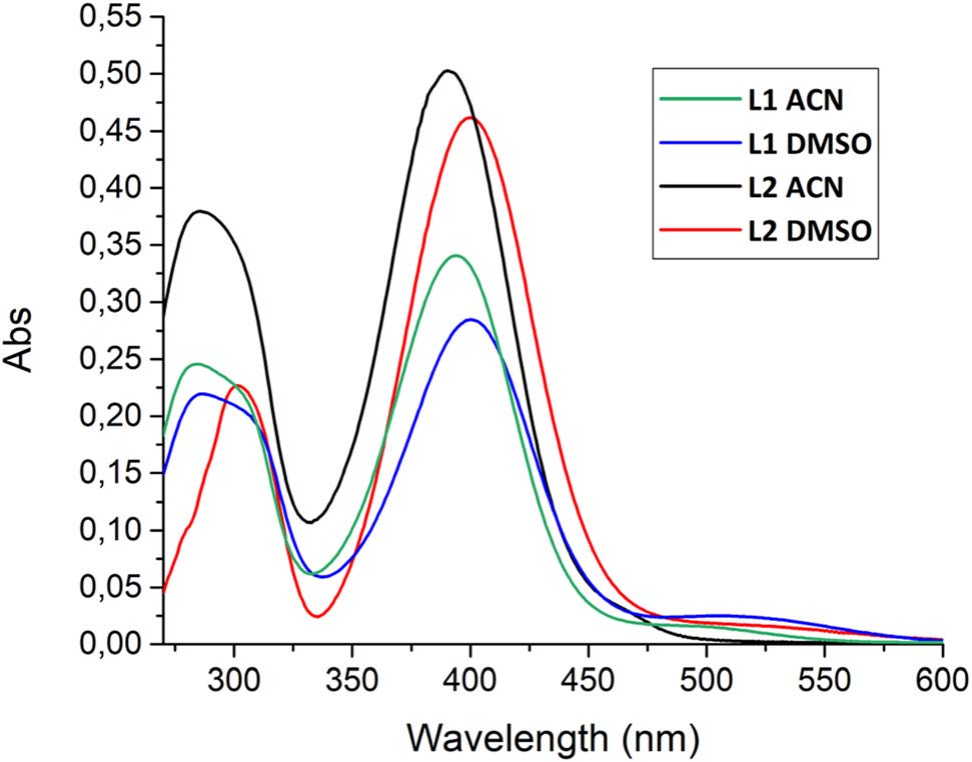
Absorption spectra of L1 in ACN (green) and DMSO (blue) and L2 in ACN (black) and DMSO (red).

Since L1 and L2 are very poorly soluble in water, the photophysical properties of both ligands were characterized in DMSO and in a mixture of ACN/DMSO (99/1 v/v); the choice to operate with 1% of DMSO in the latter case is again due to the low solubility of the ligands. In ACN, the absorption spectra of both ligands (Fig. 5, black and green lines) show, along with an absorption band at 280 nm largely due to the squaramide moieties,^38^ only a band in the 350–450 nm region attributed to the coumarin moieties (for L2, *λ*_max_ = 390 nm and *ε* = 50 000 M^−1^ cm^−1^, for L1, *λ*_max_ = 390 nm and *ε* = 33 000 M^−1^ cm^−1^). In DMSO, the absorption spectra of the two receptors (Fig. 5, red and blue lines) show similar behaviour (for L2, *λ*_max_ = 400 nm and *ε* = 45 000 M^−1^ cm^−1^, for L1, *λ*_max_ = 400 nm and *ε* = 28 000 M^−1^ cm^−1^), except for the appearance of a shoulder at around 500 nm that can be attributed to the presence of a small percentage of the deprotonated species in this solvent (see discussion below).

The emission spectra of L1 and L2 (Fig. S1), recorded upon excitation at 400 nm, show in both solvents an emission band peaking at 470 nm that is much weaker (*Φ*_fl (ACN)_ < 0.01) with respect to the one of the parent (7-(amino)-4-trifluoromethyl)coumarin (*Φ*_fl_ = 0.57 and 0.48 in ACN and DMSO, respectively).^45^

Similar effects have been previously observed and explained in terms of the occurrence of an oxidative photoinduced electron transfer (PET) effect from the higher SOMO of the excited state of the coumarin unit to the LUMO of the ground state of the squaramide group. The weak emission properties of L1 and L2 also could be explained by a non-radiative twisted intramolecular charge transfer (TICT) state, which is an effect that has been reported for many 7-amminocoumarines in polar solvents.^46^

The lifetimes were found to be 4.9 ns for both L1 and L2 in both solvents (Fig. S2).

### Acid–base behaviour

**Fig. 6:**
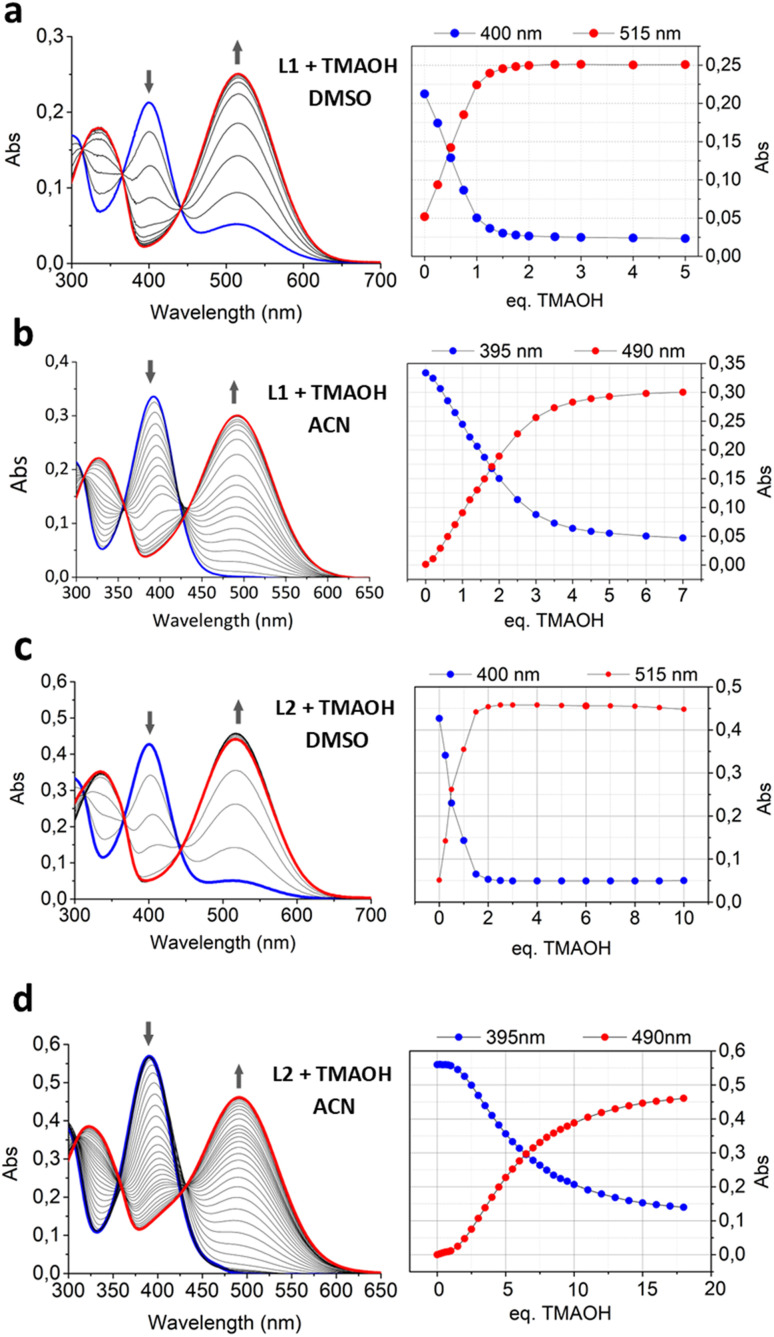
UV-Vis titrations of (a) L1 in DMSO, (b) L1 in ACN, (c) L2 in DMSO, and (d) L2 in ACN upon the gradual addition of TMAOH. [L1] = [L2] = 1 × 10^−5^ mol dm^−3^. On the left, the trend of the maximum absorption intensity at 400 nm (blue spots) and 515 nm (red spots) in DMSO and 395 nm (blue spots) and 490 (red spots) in ACN. In all the experiments, the blue line refers to the spectrum of the free ligand and the red line to the spectrum registered after the last addition of the base.

The strong capability to bind anionic species of aryl-squaramides has been explained in terms of their acidity, as proposed by Sandler *et al.*^47^ who was first to furnish scientific data to support this theory. The acidic properties of aryl-squaramides strongly depend on the specific physico-chemical properties of solvents such as the polarity, dielectric constant and donor number. With this in mind, the acid–base behaviour of L1 and L2 was studied through spectrophotometric titrations in ACN and DMSO by adding increasing amounts of different bases, chosen for their different basic force and characteristic (amines *vs.* hydroxide), *i.e.* 1,5-diazabiciclo(5.4.0)undec-7-ene (DBU), *N*,*N*-diisopropylethylendiamine (DIPEA) and the ionic tetramethylammonium hydroxide (TMAOH) (Fig. 6, S3 and S4).

The absorption spectra acquired during the titrations of L1 and L2 with TMAOH show the decrease of the absorption intensity relative to the coumarin band in both solvents (*λ*_max(DMSO)_ = 400 nm, *λ*_max(ACN)_ = 390 nm), along with the appearance of a new band (*λ*_max(DMSO)_ = 515 nm, *λ*_max(ACN)_ = 490 nm) that can be ascribed to the anionic form of the ligand, in which the squaramide NH group linked to coumarin is deprotonated (*vide infra*) (Fig. 6). In DMSO, the deprotonation band also is clearly visible in the absence of the base (blue lines in Fig. 6a and c). This means that in this solvent, the ligand is sufficiently acidic to be present in both protonated and deprotonated species in equilibrium between them. Another observation that supports the strong acidity of L1 and L2 in DMSO is the result of the same titrations carried out in ACN (Fig. 6b and d). In this solvent, following the trend of absorption at 390 and 490 nm, a plateau is obtained only after the addition of an excess of TMAOH (over 5 eq. for L1 and 10 eq. for L2). Meanwhile, in DMSO (Fig. 6a and c), the quantitative deprotonation requires only one and two equivalents for L1 and L2, respectively. In any case, the necessary equivalents to reach the plateau suggest that only one NH of the squaramide gets deprotonated for each moiety. As above, the difference in the acidity of the squaramide in the two solvents can be ascribed to the higher polarity of DMSO that favors the stabilization of the deprotonated charged species better in DMSO than in ACN.

The same experiments were carried out with DBU (Fig. S3) and DIPEA (Fig. S4) in DMSO and ACN. Similarly, the results of the titrations performed in ACN showed that the full deprotonation was reached after the addition of an excess of DBU (about 5 eq.) for both L1 and L2 (Fig. S3b and d).

Instead, in DMSO, the full deprotonation is reached after the addition of 0.5 eq. and 1.0 eq. of DBU for L1 and L2, respectively (Fig. S3a and c). The behaviour observed with DBU in DMSO suggests a possible formation of L–H⋯L^−^ stable homo-conjugates, where the formed anion is hydrogen bonded to its respective neutral species, and part of a N–H⋯N^−^ intramolecular hydrogen bond network in the ditopic L2. The same did not occur with TMAOH. This is probably due to the instauration of hydrogen bonding between the base and the squaramide groups, as confirmed by the fact that L1 and L2 reached saturation upon the addition of 1 and 2 eq. of base, respectively. Data obtained by titrating the two ligands with DIPEA show the non-capability of this base to deprotonate the squaramide N–H groups, showing only a weak increase of the band relative to the deprotonated form. This is probably due to its lower basicity and higher steric hindrance (Fig. S4).

The cause of the higher acidic character of the squaramide groups in DMSO compared to that in ACN can be explained by the combination of the greater donor number (29.8 kcal mol^−1^ for DMSO *vs.* 14.1 for ACN) and dielectric constant (47 for DMSO *vs.* 36 for ACN), allowing for the stabilization of the H^+^ cation (DMSO-H^+^), while also favouring the ionization of covalent bonds.^48^

Although L1 and L2 showed different acidities in the two solvents, attempts to quantify their acidity constants failed. This is probably because the constant values were too high to be safely evaluated by spectrophotometric titrations, denoting the high tendency toward deprotonation of the coumarin-squaramide moiety.

To further investigate the acidic behaviour of the two receptors, ^1^H NMR studies were performed by titrating L1 and L2 with TMAOH in DMSO-*d*_6_ + 0.5% of D_2_O. The choice to operate with a small amount of deuterated water is due to the attempt to minimize the quantity of D_2_O that inevitably would enter the DMSO-*d*_6_ solution, affecting the results. Fig. S5 shows the spectra of the two ligands (Fig. S5a for L1 and Fig. S5b for L2) registered in both DMSO-*d*_6_ and DMSO-*d*_6_ + 0.5% of D_2_O. As expected, one of the differences between the two spectra is the disappearance of the squaramide signals (H5 and H6) justified by the typical rapid exchange of acidic protons. The other signals remained virtually unchanged, showing only a slight up-field shift of all resonances in the presence of D_2_O, suggesting that the system is not significantly perturbed by the presence of water.

Data obtained during the titration of L1 (Fig. S6a) and L2 (Fig. S6b) with TMAOH in DMSO-*d*_6_ + 0.5% of D_2_O show an upfield shift of all the aromatic coumarin signals (H7, H8, H9, H10), while no significant effects on the phenyl signals were observed (H1, H2, H3, for both L1 and L2). As previously suggested by the UV-Vis experiments, in this case, the deprotonation also seems to totally occur after the addition of 1.0 eq. of the base for L1. Meanwhile, in the case of L2, the upfield shift of the signals is visible for up to 2 eq. of added titrant. Taken together, these results confirm that the deprotonation involves the N–H groups of the squaramide functional unit that is closer to the coumarin moiety, for both ligands.

### Interaction with NSAIDs

**Fig. 7:**
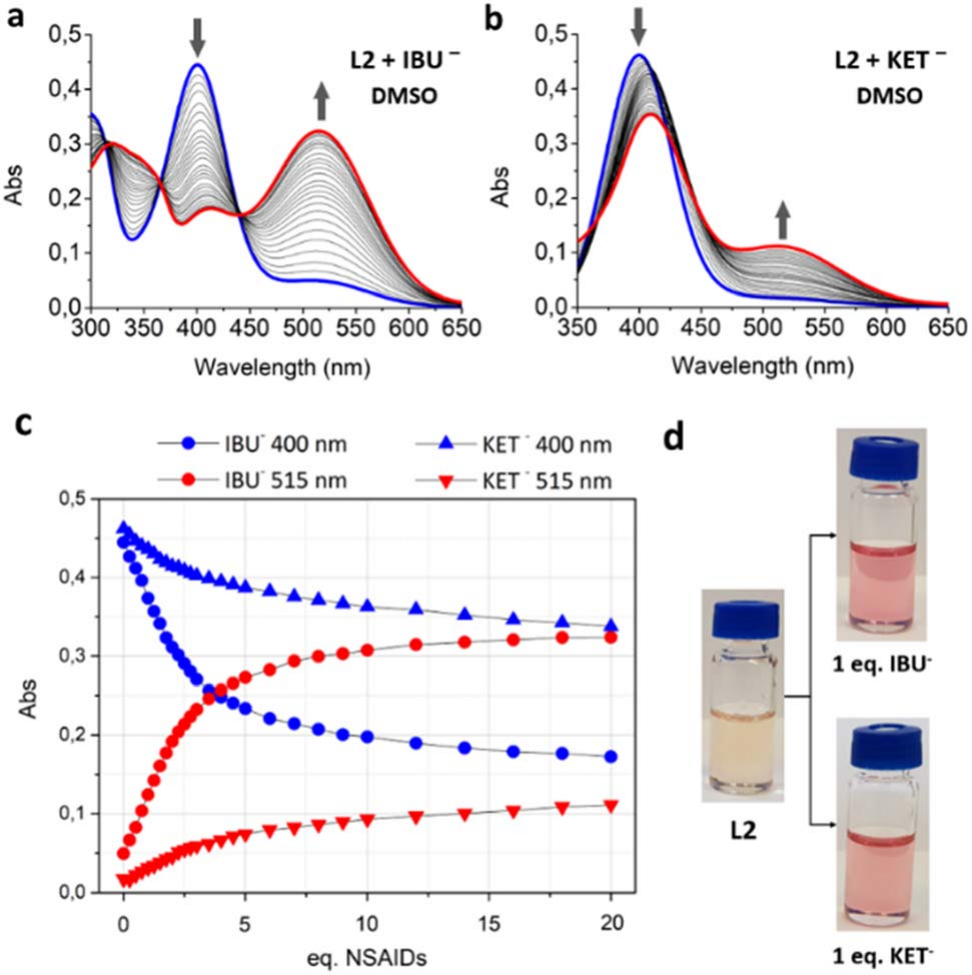
UV-Vis titrations of L2 in DMSO with (a) IBU^−^ and (b) KET^−^, blue lines refer to the spectrum of the free ligands and red lines to the spectrum registered after the last addition of the anion; (c) the trend of the maximum absorption intensity at 400 nm (blue spots) and 515 nm (red spots). [L2] = 1 × 10^−5^ mol dm^−3^; and (d) colour change after the addition of 1 eq. of IBU^−^ and KET^−^ to a 1 × 10^−5^ mol dm^−3^ DMSO solution of L2.

The interaction of L1 and L2 with NSAIDs has been studied by spectrophotometric, ^1^H-NMR and ESI-MS measurements and DFT calculations. UV-Vis titrations were conducted by adding increasing amounts of BzO^−^, IBU^−^, NPX^−^ and KET^−^ anions as sodium salts to DMSO solutions of L1 and L2. Upon the addition of NSAIDs, no variations in the emission intensity or lifetime were observed. On the other hand, significant changes were observed in the absorption spectra. The experiments performed on L1 with all the selected carboxylate drugs showed a spectral pattern very similar to that observed during the deprotonation processes described above (Fig. S7a–d). In other words, the addition of increasing amounts of the guests to the DMSO solution of the ligand caused a decrease of the band at 400 nm, along with a growth of the signal at 515 nm. The same behaviour was observed for L2 (Fig. 7a for IBU and Fig. S7e and d for BzO^−^ and NPX^−^, respectively) with the only exception of KET^−^ (Fig. 7b), whose addition caused a smaller decrease of the band at 400 nm and a concurrent redshift of the absorption maximum from 400 to 410 nm, together with a slight increase of the deprotonation band at 515 nm. The overall trend of the absorption intensities at 400 and 515 nm observed upon addition of all the drugs (Fig. 7c) is similar to that observed for the titration of L2 with TMAOH (Fig. 6c). Meanwhile, the trend is significantly different in the case of KET^−^ (Fig. 7c).

**Fig. 8:**
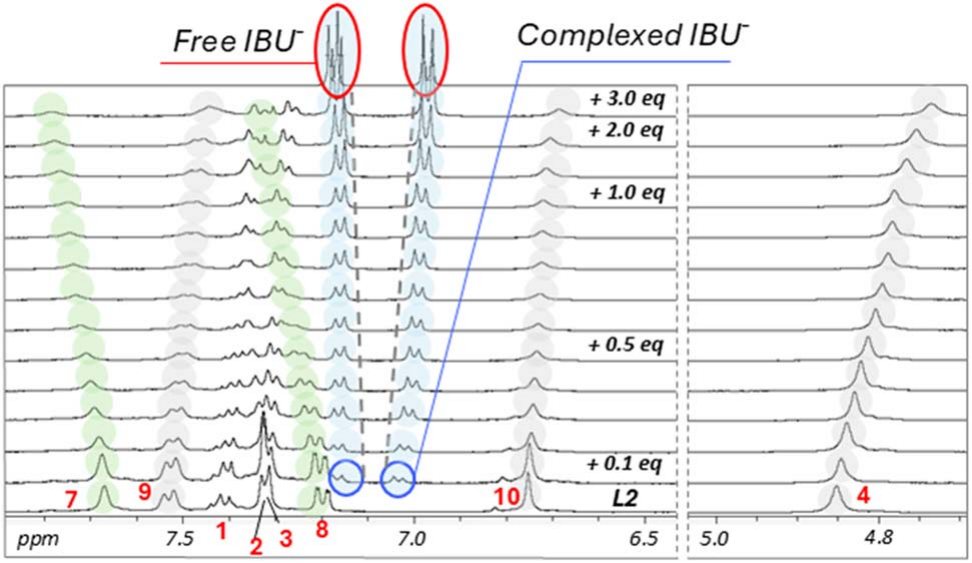
^1^H NMR spectra of L2 registered in DMSO-*d*_6_ + 0.5% of D_2_O upon the gradual addition of sodium ibuprofen. [L2] = 7.5 × 10^−3^ mol dm^−3^. The upper spectrum shows the signal of free sodium ibuprofen registered in DMSO-*d*_6_ + 0.5% of D_2_O.

To understand the different behaviour of KET^−^ with respect to the other drugs, the same titrations were performed with ^1^H-NMR spectroscopy, comparing the results obtained with IBU^−^ and KET^−^. The titration that was carried out by adding IBU^−^ to a solution of L2 showed significant shifts of all resonances for both host and guest (Fig. 8). In particular, the H4, H9 and H10 resonances underwent upfield shifts in a manner that was comparable to what was observed in the ^1^H-NMR titration of L2 with TMAOH (Fig. S6b). In the case of H7 and H8, different from the titration with the base, a downfield shift for both signals was observed.

Moreover, looking at the resonances of IBU^−^ (light blue circles in Fig. 8) in the spectrum registered after the first addition of the guest (+0.1 eq.), the two signals are closer to each other with respect to the same resonances in the free IBU^−^ (top spectrum, red circles), while further additions of the guest shifted the resonances towards those of the free form of the anion.

These findings suggest that in solution, both deprotonation and interaction phenomena can coexist.

However, by combining the UV-Vis and ^1^H-NMR results obtained in DMSO with IBU^−^, it can be supposed that the equilibrium tends toward the deprotonation process of L2 rather than the formation of a complex.

In the ^1^H-NMR titration performed with KET^−^, the data interpretation is more complicated due to the overlapping of the peaks of the anion with those of the ligands (Fig. S8). Certainly, also in this case, it is possible to observe several differences between this titration and the one carried out with the TMAOH base (Fig. S6b). H4 and H10, as in the experiments performed with TMAOH and IBU^−^, underwent an upfield shift. Analogous to what was observed for IBU^−^, H7 and H8 moved downfield. Furthermore, resonances related to the phenyl group were also significantly influenced, with H1, H2 and H3 showing a slight but evident upfield shift. These findings, together with the fact that the system seemed to reach saturation after 1.0 eq. of KET^−^ (only slight shifts of the resonances are visible between the addition of 1 and 2 eq. of KET^−^), suggest a possible cooperation of the two squaramide groups of L2 upon anion complexation.

The addition of both guests (Fig. 7d and S7c) and bases (Fig. S7c) to DMSO solutions of L1 and L2 is visible to the naked eye *via* a colour change of the solution from pale orange to bright pink.

**Fig. 9:**
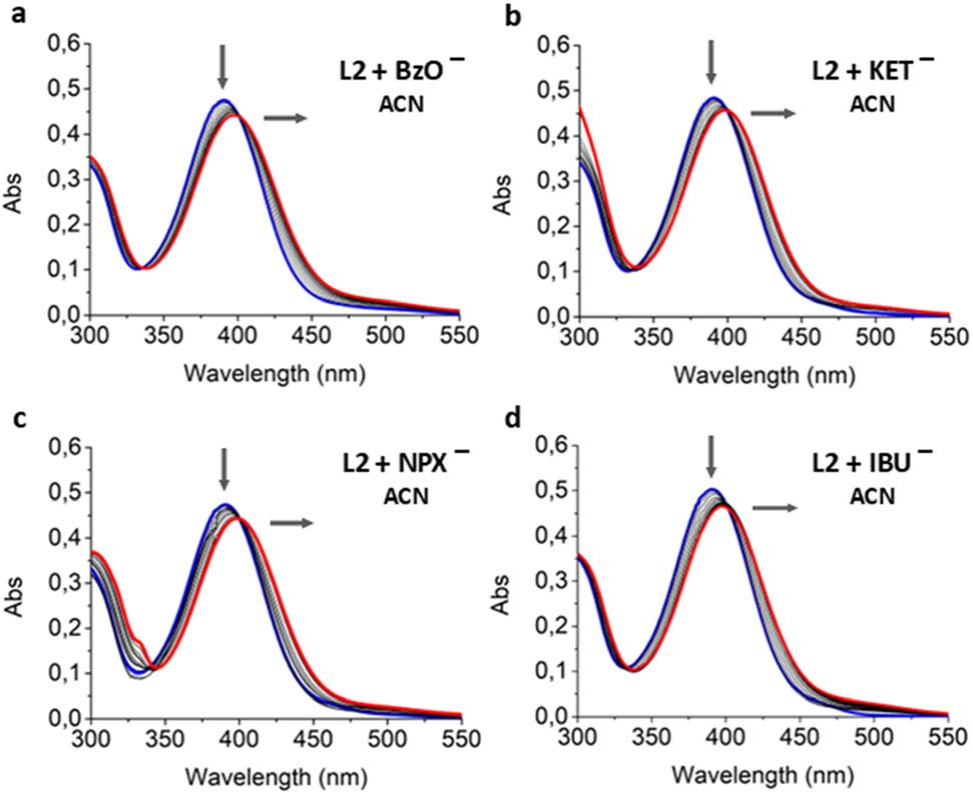
UV-Vis titrations performed in ACN of L2 with the gradual addition (0–10 eq.) of (a) BzO^−^, (b) KET^−^, (c) NPX^−^, (d) IBU^−^ [L2] = 1 × 10^−5^ mol dm^−3^. In all of the experiments, blue lines refer to the spectrum of the free ligands and red lines to the spectrum registered after the last addition of the anion.

The interaction of L1 and L2 with sodium salts of NSAIDs and benzoate was also spectrophotometrically investigated in ACN. Results obtained from the titrations of both ligands with all the selected carboxylates showed different behaviors (Fig. 9 and S9) compared to what was observed during the UV-Vis titrations with the base in the same solvent (Fig. 6b and d). In these experiments, the addition of an increasing amount of guest caused a shift of the absorbance peak at 390 nm towards longer wavelengths with a very slight increase of the band at 500 nm, which is attributable to the deprotonation of the ligands. However, the different spectroscopic outcome of the titrations performed in the two solvents suggests that the formation of the host–guest complex in ACN is more evident. Given that NSAIDs and benzoate are weak bases, it would be appropriate to hypothesize that L1 and L2 allow for an equilibrium between the complex formation and deprotonation process in the presence of carboxylates. In such a case, there would be a strong tendency toward deprotonation in DMSO, while host–guest interaction is favored in ACN.

The UV-Vis titrations were processed to evaluate the host–guest association constant values (Table 2) with the BindFit program using UV-Vis titration data obtained in ACN.^49^

**Table 2:** Association constants (log *K*_ass_) found for L2 obtained by fitting the UV-Vis titration data performed in ACN

Association constant (log *K*_ass_)	
Adduct	L2
[L2-KET]^−^	4.88(6)^a^
[L2-NPX]^−^	4.77(6)
[L2-IBU]^−^	4.88(6)
[L2-BzO]^−^	5.16(4)

a Values in parenthesis are standard deviations on the last significant figure.

Unfortunately, the algorithm was not able to fit the titration of L1 for either the 1 : 1 or the 2 : 1 model. Instead, for L2, it was possible to obtain association constant values and they are listed in Table 2. Looking at the log *K*_ass_ values, no selectivity for a specific anion was found.

To better understand the stoichiometry of possible adducts of L1 and L2 with NSAIDs, ESI-MS experiments were performed. To completely avoid the presence of DMSO, saturated solutions of L1 and L2 were prepared in pure ACN alone and in the presence of IBU^−^, NPX^−^, KET^−^ and BzO^−^, and the results obtained are listed in Table 3. The peaks corresponding to the deprotonated ligands ([L1-H]^−^ = 413 in Fig. S10 and [L2-H]^−^ = 749 in Fig. S11) were easily detected.

**Table 3:** Results of the ESI-MS experimental results (*m*/*z*)

Anion	MW	L1	L2
—		[L1-H] = 413	[L2-H] = 749
Cl^−^a	35.45	[L1_2_-Cl]^−^ = 863	[L2-Cl]^−^ = 785
NO_3_^−^a	62.00	[L1-NO_3_]^−^ = 476	[L2-NO_3_]^−^ = 812
		[L1_2_-NO_3_]^−^ = 890	—
KET	253.08 b	[L1-KET]^−^ = 667	[L2-KET]^−^ = 1033
		[L1_2_-KET]^−^ = 1081	—
NPX^−^	229.08 b	[L1_2_-NPX]^−^ = 1057	[L2-NPX]^−^ = 979
IBU^−^	205.28 b	[L1_2_-IBU]^−^ = 1033	[L2-IBU]^−^ = 955

a These anions have not been added and.

b Detected with this experiment.

The mass spectrum of L1 showed the tendency to form an adduct with a 2 : 1 stoichiometry ligand to the anion ([**L1**_**2**_-Anion]^−^) (Fig. S10), while only an adduct with a 1 : 1 stoichiometry ([L2-Anion]^−^) is visible for L2 (Fig. S11).

Table 3 also shows the results of the analysis carried out on the ACN solutions of the two ligands after the addition of KET^−^ (Fig. S12 for L1 and Fig. S15 for L2), NPX^−^ (Fig. S13 for L1 and Fig. S16 for L2) and IBU^−^ (Fig. S14 for L1 and Fig. S17 for L2).

These findings confirm the ability of the new coumarin-squaramide based receptors to effectively form host–guest complexes with NSAIDs.

### DFT calculations

In host–guest chemistry, DFT calculations are usually carried out to obtain an atomistic view of the groups involved in the non-covalent interactions.^50,51^ In this work, we have used such methodology to provide host–guest models whose calculated properties are in agreement with the experimental data obtained for the complexes between L1 and IBU^−^, reported and discussed above.

**Fig. 10:**
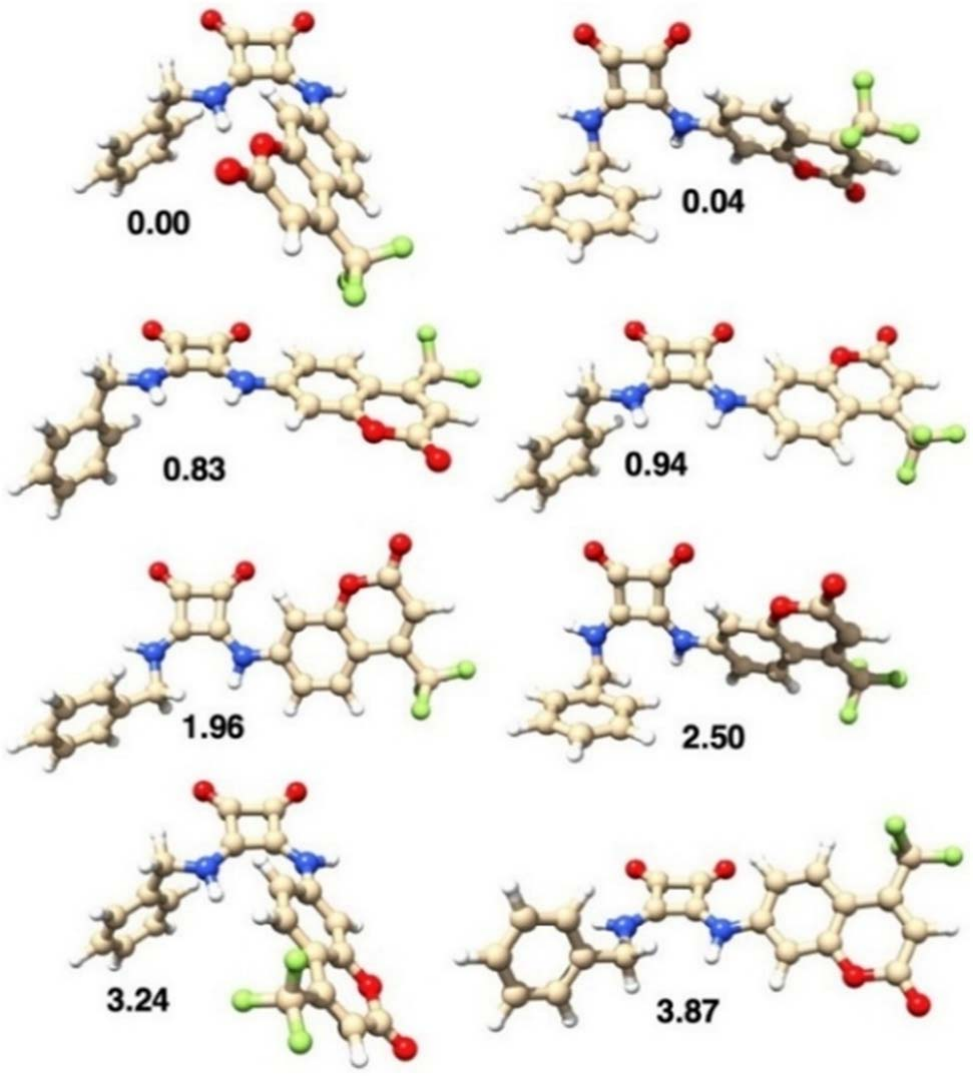
Structure of the conformers of L1 obtained by DFT calculations in DMSO and listed in order of increasing relative values of the standard Gibbs free energy (in kJ mol^−1^).

The structures of L1 and its conformers found by DFT calculations, in both DMSO and ACN solvents, are reported in Fig. 10 and S18, respectively. The conformation found in the L1·DMSO crystal structure corresponds to the energy minimum (the first conformer) in ACN, and to the fourth (more stable) conformer in DMSO. Interestingly, in DMSO, the four more stable conformations differ by less than 1 kJ mol^−1^, indicating that these structures are all accessible in such solution (Fig. 10). Moreover, their calculated UV absorption spectra are almost superimposable (Fig. S19), indicating that all conformations provide similar absorption spectra. In ACN, the energy difference found among the conformers is generally higher (Fig. S18), but the first four more stable conformers lie within 3 kJ mol^−1^.

**Fig. 11:**
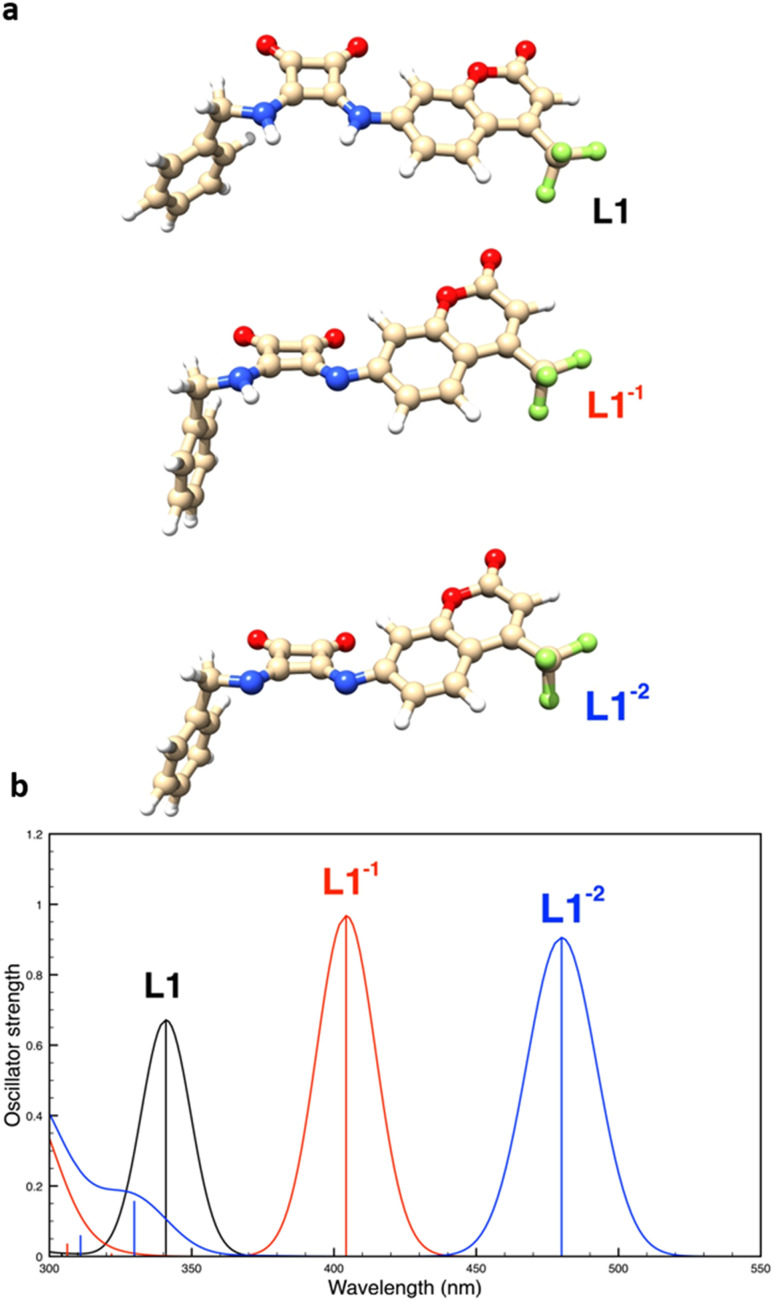
(a) Structure of the neutral and anionic species of the fourth conformer of L1 obtained by the DFT calculations in DMSO. (b) Corresponding UV absorption spectra obtained by the TD-DFT calculations in DMSO.

The structures of the neutral and anionic forms of the fourth conformer of L1 in DMSO, as well as that of the corresponding calculated absorption spectra, are shown in Fig. 11. In agreement with the experimental findings, the obtained results show that each deprotonation of the ligand produces a red shift of the lowest energy absorption band.

**Fig. 12:**
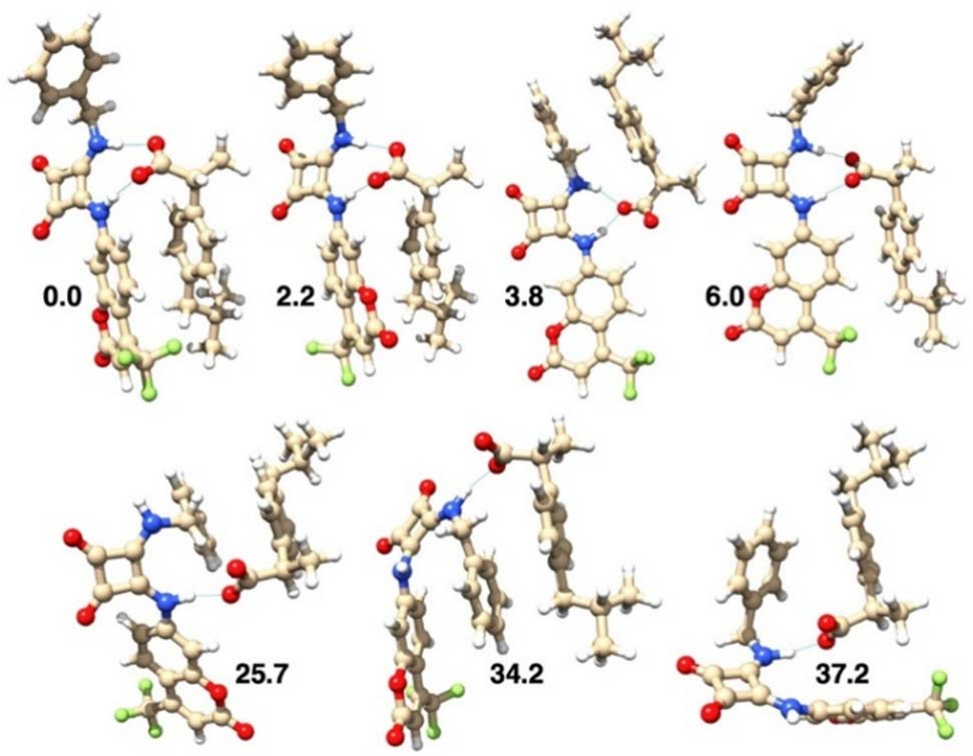
Structures of the isomers of the binding complexes between L1 and IBU^−^, obtained by the DFT calculations in DMSO and listed in order of increasing relative values of the standard Gibbs free energy (in kJ mol^−1^).

The structures of seven different isomers of the binding complexes between L1 and IBU^−^, in both DMSO and ACN solvents, are reported in Fig. 12 and S20, respectively. In both solvents, the most stable isomer has a conformation analogous to that found in the L1·DMSO solid state structure (Fig. 2). However, both oxygen atoms (in DMSO) and only one oxygen atom (in ACN) of the carboxylate group are involved in the H-bonds with the hydrogen atoms of the two NH groups, appearing in the most stable isomer.

It is interesting to note that in all cases, but particularly in the more stable species, stacking interactions occur between the aromatic ring of IBU^−^ and one of the two aromatic groups attached to the squaramide unit of L1.

The calculated binding energy, considering the most stable conformers of IBU^−^, L1 and of the L1-IBU^−^ complex, is −42.5 and −38.9 kJ mol^−1^, in DMSO and ACN, respectively.

**Fig. 13:**
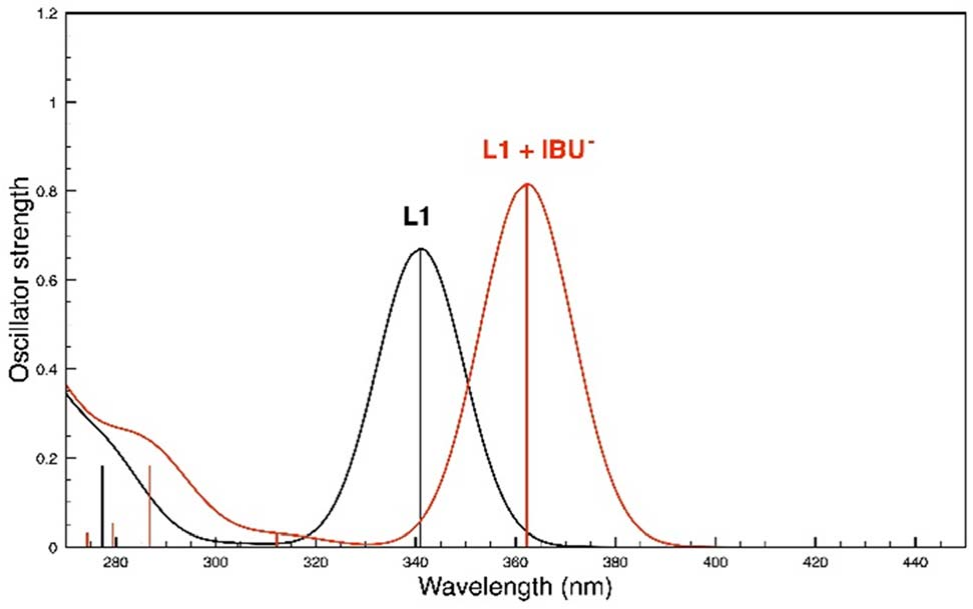
UV absorption spectra obtained by the TD-DFT calculations in DMSO of L1 and of the more stable complex between L1 and IBU^−^.

The calculated absorption spectra of L1 and of the most stable isomer of the L1-IBU^−^ complex are reported in Fig. 13. It is interesting to note that, although there is no net deprotonation of the NH groups involved in the H-bonds with IBU^−^, there is a reasonable red shift of the lowest energy absorption band following the formation of the host–guest complex.

The results obtained allow us to conclude that the DFT-calculated structures and spectra provide reliable models for the species present in solution, and support the interpretation of the experimental data.

## Experimental

### Synthesis of L1 and L2

The solvents were RP grade, unless otherwise indicated. All reactions involving moisture-sensitive reagents were carried out under nitrogen atmosphere using standard vacuum line techniques and glassware that was flame-dried before use.

### 3-Ethoxy-4-((2-oxo-4-(trifluoromethyl)-2*H*-chromen-7-yl)amino)cyclobut-3-ene-1,2-dione (3)

To a stirred solution of 1 (890 mg, 5.23 mmol) and zinc trifluoromethanesulfonate (160 mg, 0.440 mmol) in 20 ml di EtOH at room temperature was added 20 ml ethanol solution of 7-amino-4-(trifluoromethyl)coumarin (1.20 g, 5.24 mmol). The mixture was stirred overnight at room temperature.

The yellow precipitate formed was filtered and washed with ethanol, yielding 3 as a yellow solid (1.41 g, 76%).

^1^H-NMR (600 MHz, DMSO-*d*_6_): *δ* (ppm) = 1.46 (3H, t, *J* = 7.1 Hz), 4.83 (2H, q, *J* = 7.1 Hz), 6.93 (1H, s), 7.48 (1H, dd, ^1^*J* = 8.9 ^2^*J* = 2.4 Hz), 7.60 (1H, d, *J* = 1.6 Hz), 7.71 (1H, dd, ^1^*J* = 8.7 ^2^*J* = 1.6 Hz), 11.2 (1H, s) (Fig. S21). ^13^C-NMR (150 MHz, DMSO-*d*_6_): *δ* (ppm) = 16.1, 70.7, 107.0, 109.0, 115.0 (br q, ^3^*J*_CF_ = 5.4 Hz), 116.6, 122.1 (q, ^1^*J*_CF_ = 275.4 Hz), 126.3, 139.6 (q, ^2^*J*_CF_ = 32.7 Hz), 143.1, 155.4, 159.0, 169.8, 180.3, 185.0, 187.6 (Fig. S22).

### Synthesis of 3-(benzylamino)-4-((2-oxo-4-(trifluoromethyl)-2*H*-chromen-7-yl)amino)cyclobut-3-ene-1,2-dione (L1)

To a stirred suspension of 3 (100 mg, 0.283 mmol) in ethanol (50 ml) was added 4 (31 µL, 0.283 mmol). Upon the addition of the amine, the suspension changes from a yellow color to intense red. The suspension was stirred overnight at room temperature. The day after, the reaction mixture was immersed in an ice bath to promote the complete precipitation of the product. Then, the resulting solid was filtered and the precipitate was purified in good yields by crystallization from hot ethanol, yielding L1 as an orange solid (83.2 mg, 71%).

^1^H-NMR (400 MHz, DMSO-*d*_6_): *δ* (ppm) = 4.84 (2H, d, *J* = 6.2 Hz), 6.87 (1H, s), 7.31 (1H, dd, ^1^*J* = 8.8 ^2^*J* = 2.3 Hz), 7.33–7.38 (1H, m), 7.39–7.44 (4H, m), 7.67 (1H, dd, ^1^*J* = 8.7 ^2^*J* = 2.0 Hz), 7.81 (1H, s), 8.17 (1H, s), 10.15 (1H, s) (Fig. S23). ^13^C-NMR (100 MHz, DMSO-*d*_6_): *δ* (ppm) = 47.8, 105.8, 107.9, 114.0 (br q, ^3^*J*_CF_ = 5.6 Hz), 115.6, 122.1 (q, ^1^*J*_CF_ = 277.9 Hz), 126.5, 128.1, 128.2, 129.2, 138.5, 139.8 (q, ^2^*J*_CF_ = 31.2 Hz), 143.9, 155.8, 159.1, 162.9, 170.1, 180.7, 185.5 (Fig. S24).

Elemental analysis for C_21_H_13_F_3_N_2_O_4_: calcd C 60.88, H 3.16, N 6.76; found C 60.8, H 3.2, N 6.7.

(ESI): *m*/*z* calcd. for C_21_H_13_F_3_N_2_O_4_: 414.08; found: 413.08 [M–H]^−^.

### 4,4′-((1,3-Phenylenebis(methylene))bis(azanediyl))bis(3-((2-oxo-4-(trifluoromethyl)-2*H*-chromen-7-yl)amino)cyclobut-3-ene-1,2-dione) (L2)

To a stirred suspension of 3 (100 mg, 0.283 mmol) in ethanol (50 ml) was added 5 (19 µL, 0.142 mmol). Upon the addition of the amine, the suspension turns from a yellow color to intense red. The suspension was stirred overnight at room temperature. The day after, the reaction mixture was immersed in an ice bath to promote the complete precipitation of the product. Then, the resulting solid was filtered and the precipitate was purified in good yields by crystallization from hot ethanol, yielding L2 as a red solid (95.0 mg, 89%).

^1^H-NMR (400 MHz, DMSO-*d*_6_): *δ* (ppm) = 4.87 (4H, d, *J* = 5.8 Hz), 6.81 (2H, s), 7.24 (2H, dd, ^1^*J* = 8.8 ^2^*J* = 2.3 Hz), 7.38–7.30 (3H, m), 7.43 (1H, t, *J* = 7.5 Hz), 7.56 (2H, d, *J* = 8.1 Hz), 7.73 (2H, s), 8.26 (2H, br s), 10.21 (2H, br s) (Fig. S25).

^13^C-NMR (100 MHz, DMSO-*d*_6_): *δ* (ppm) = 47.6, 105.7, 107.7, 113.8 (br q, ^3^*J*_CF_ = 5.6 Hz), 115.6, 126.2, 126.3, 127.1, 129.6, 139.4, 139.8 (q, ^2^*J*_CF_ = 32.7 Hz), 144.0, 155.7, 159.0, 163.0, 170.3, 180.8, 185.6 (Fig. S26).

Elemental analysis for C_36_H_20_F_6_N_4_O_8_: calcd. C 57.61, H 2.69, N 7.46; found C 57.4, H 2.8, N 7.5.

(ESI): *m*/*z* calcd. for C_40_H_42_N_6_O_6_S: 750.12; found: 749.11 [M–H]^−^.

### Synthesis of crystals

L1 (5 mg) was dissolved in hot DMSO (1 ml) and then the solution was cooled to room temperature, allowing a slow evaporation of the solvent. After 3 days, orange crystals suitable for X-ray analysis appeared.

### X-ray crystallography

SCXRD data of L1·DMSO were collected on a Bruker Apex-II diffractometer equipped with a CCD detector (*T* = 100 K, Cu-Kα radiation (*λ* = 1.54178 Å)). Data were collected with the APEX2 software,^52^ while data integration and reduction were performed with the Bruker SAINT software.^53^ The crystal structure was solved using the SIR-2004 package^54^ and refined by full-matrix least squares against F2, using all data (SHELXL-2018/3).^55^ All the non-hydrogen atoms of the structure were refined with anisotropic displacement parameters. The hydrogen atoms were found in the Fourier difference maps and their coordinates were freely refined, while their thermal parameters were set in accordance with that of the atoms to which they are bonded. Geometrical calculations were performed by PARST97 (ref. 56) and molecular plots were produced by the program CCDC Mercury (v. 2022.3.0).^57^

Crystallographic data and refinement parameters are reported in Table 4.

**Table 4:** Crystal data and refinement parameter of L1·DMSO

	L1·DMSO
Empirical formula	(C_21_H_13_F_3_N_2_O_4_)(C_2_H_6_SO)
Formula weight	492.46
*T* (K)	100
Crystal system, space group	Orthorhombic, *Pbca*
Unit cell dimensions (Å, °)	*a* = 20.5435(5)
	*b* = 7.9840(2)
	*c* = 26.6642(7)
*V* (Å^3^)	4373.4(2)
*Z*, d_calc_(g cm^−3^)	8, 1.496
*µ* (mm^−1^)	1.904
*F*(000)	2032
Reflections collected/unique/*R*_int_	50 298/3977/0.1168
Data/parameters	3977/364
Final *R* indices [*I* > 2*σ*(*I*)]	0.0456/0.1193
*R* Indices all data	0.0635/0.1324
GOOFs	1.020

### Computational details

DFT calculations, with full geometry optimization, have been carried out on the ligand L1, as well as on its complex with ibuprofen, by using the ωB97X-D functional^58^ and the 6-31+G(d,p) basis set.^59,60^ The effect of the implicit DMSO and ACN solvents was evaluated using the SMD method.^61^ This level of theory has been successfully applied in a recent study on squaramide derivatives.^47^

Vibrational frequency calculations within the harmonic approximation were performed to confirm that the optimized geometries represented a minimum in the potential energy surface, and to calculate the relative standard Gibbs free energy values. Time-dependent (TD) DFT calculations^62,63^ were performed on all the species found to calculate their UV-Vis absorption spectra. All calculations were performed using the Gaussian 16 program package.^64^

### NMR studies

^1^H and ^13^C NMR spectra were recorded on a Bruker Avance 400 instrument (Bruker Italia, Milan, Italy), operating at 400.13 and 100.61 MHz, respectively, and equipped with a variable temperature controller. The temperature of the NMR probe was calibrated using 1,2-ethanediol as a calibration sample. Chemical shifts (*δ* scale) are presented in ppm and referenced by the residual solvent peak. coupling constants (*J* values) are given in hertz (Hz). ^1^H–^1^H and ^1^H–^13^C correlation experiments were performed to assign the signals.

^1^H-NMR titrations were performed at 298.1 K mixed in DMSO-*d*_6_ to prevent solubility issues. The small amount of D_2_O (0.5%) was added to avoid the uncontrolled absorption of water by DMSO-*d*_6_ during the measurements, keeping a fixed amount of water in solution.

### Spectrophotometric and fluorescence measurements

Absorption spectra were recorded using a double beam spectrophotometer UV/Vis PerkinElmer Lambda 850+. Emission and excitation spectra were recorded using a spectrofluorometer PerkinElmer LS55.

The fluorescence QYs were measured against quinine sulphate in H_2_SO_4_ (*Φ*_fl_ = 0.54). Fluorescence lifetime measurements were performed using a spectrofluorometer (Edinburgh Analytical Instruments FLS920), equipped with a time-correlated single-photon counting device and a 405 nm pulsed diode laser.

All spectroscopic measurements were conducted using spectroscopy-grade solvents purchased from Merck and using 1 cm optical path quartz cuvettes purchased from Hellma.

Association constants were obtained by using the open-source program Bindift program.

### ESI-MS measurements

The mass spectra were recorded using a Xevo G2-XS QTof system by Waters. The solutions were directly injected into the ESI-QTof system in negative polarity mode.

## Conclusions

Two new receptors (L1 and L2) for NSAIDs were easily synthesized in good yields and studied for their possible application as receptors towards NSAIDs.

IN DMSO, results of the UV-Vis and ^1^H NMR studies performed with NSAIDs showed a very similar behaviour to that observed for the titration of both ligands with TMAOH. This suggests that in the presence of the weak bases NSAIDs, L1 and L2 achieve equilibrium between complex formation and the deprotonation process, which tends toward the latter rather than the former. However, the different spectroscopic outcome of the titrations performed in ACN suggests that the formation of the host–guest complex in this solvent is more evident. Thus, both ligands admit an equilibrium where the host–guest interaction prevails over the deprotonation process.

The *K*_ass_ values (log *K*) found for the [L2-Anion]^−^ complexes with IBU^−^ (4.88), NPX^−^ (4.77), KET^−^ (4.88) and BzO^−^ (5.16) indicate the excellent capability of L2 to bind NSAIDs, even if no selectivity for a specific anion is shown.

Results of MS studies showed the tendency of L1 to form adducts with a 2 : 1 ligand-to-anion stoichiometry ([L1_2_-Anion]^−^). Meanwhile, for L2, only adducts with a 1 : 1 stoichiometry ([L2-Anion]^−^) were visible.

These findings demonstrated the ability of the two coumarin-squaramide-based receptors to form host–guest complexes with NSAIDs. Moreover, in the case of L2, the high log *K*_ass_ values and the MS results both suggest that the two squaramides cooperate to stabilize NSAIDs in the host–guest adduct. All these findings, in our opinion, can be useful for the design of increasingly sophisticated chemosensors for NSAIDs.

## Author contributions

F. I.: investigation; L. M.: investigation; D. P.: investigation, writing – original draft, writing – review and editing; G. B.: investigation, writing – original draft, P. R.: investigation, writing – original draft; L. G.: conceptualization, supervision, writing – original draft; M. F.: funding acquisition, investigation, writing – review and editing; E. M.: writing – review and editing; M. L.: investigation; L. P.: conceptualization, supervision, writing – original draft, writing – review and editing; and V. F.: supervision, writing – original draft, writing – review and editing.

## Conflicts of interest

There are no conflicts to declare.

## Supplementary Material

RA-016-D5RA08698A-s001

RA-016-D5RA08698A-s002

## Data Availability

CCDC 2501001 (L1·DMSO) contains the supplementary crystallographic data for this paper.^65^ The data supporting this article have been included as part of the supplementary information (SI). Supplementary information: NMR characterization; ^1^H-NMR, absorption and mass spectra, solid state and DFT calculation details. See DOI: https://doi.org/10.1039/d5ra08698a.
