# Supplementary material for: Synthesis and binding studies of two new coumarin-squaramide-based receptors for NSAIDs
Source: RSC Adv. 2026 Jan 19;16(5):4116–28. doi: 10.1039/d5ra08698a (PMC12814399; doi:10.1039/d5ra08698a)
Supplement: RA-016-D5RA08698A-s001 [file RA-016-D5RA08698A-s001.pdf]

Supplementary Information (SI) for RSC Advances.
This journal is © The Royal Society of Chemistry 2025

Electronic Supporting Information

Synthesis and Binding Studies of Two New Coumarin-Squaramide-Based Receptors for NSAIDs

Luca Mancini,[a] Filippo Ingargiola,[b] Giampaolo Barone,[c] Patrizia Rossi,[d] Mauro Formica,[a] Eleonora Macedi,[a] Martina Lippi,[d] Luca Giorgi,*[a] Luca Prodi*[b] Vieri Fusi *[a] and Daniele Paderni [a]

[a] *Department of Pure and Applied Sciences, University of Urbino, via Ca' Le Suore 2-4, 61029, Urbino, Italy.*

E-mail: luca.giorgi@uniurb.it; vieri.fusi@uniurb.it

[b] *Department of Chemistry "Giacomo Ciamician", Università degli Studi di Bologna, Via Selmi 2, 40126 Bologna, Italy.*

E-mail: luca.prodi@unibo.it

[c] *Department of Biological, Chemical and Pharmaceutical Sciences and Technologies (STeBiCeF), University of Palermo, Viale delle Scienze, Edificio 17, 90128 Palermo, Italy.*

[d] *Department of Industrial Engineering, University of Florence, via S. Marta 3, 5013, Florence, Italy.*

Content

1. **X-ray crystal structure details** **pag. 2**
2. **Photophysical characterization** **pag. 2**
3. **Acid-base studies** **pag. 4**
4. **Interaction with NSAIDs (UV-Vis and NMR experiments)** **pag. 8**
5. **Interaction with NSAIDs (mass experiments)** **pag. 10**
6. **DFT calculation details** **pag. 14**
7. **NMR characterization of L1 and L2** **pag. 16**

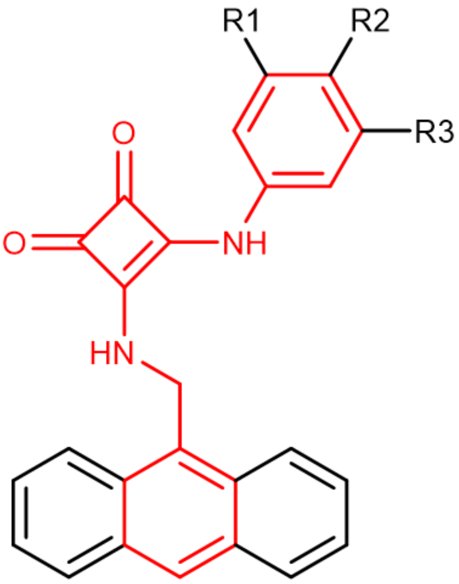

CIPYIL: R1 = R3 = CF_3; R_2 = H

CIPYOR: R1 = R3 = H; R_2 = CF_3

CIPYUX: R1 = R3 = H; R_2 = NO_2

Scheme S1. Molecular structure of the ligands in CIPYIL, CIPYOR and CIPYUX.

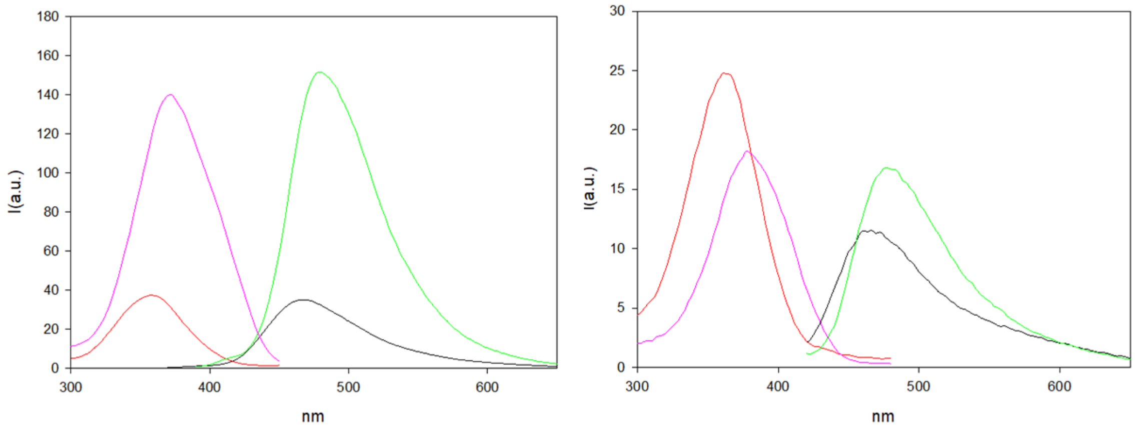

Figure S1. Emission (λ_{ex} = 400 nm) and excitation spectra (λ_{em} = 480 nm) of **L1** (right) and **L2** (left), green and pink for ACN, red and black for DMSO.

a

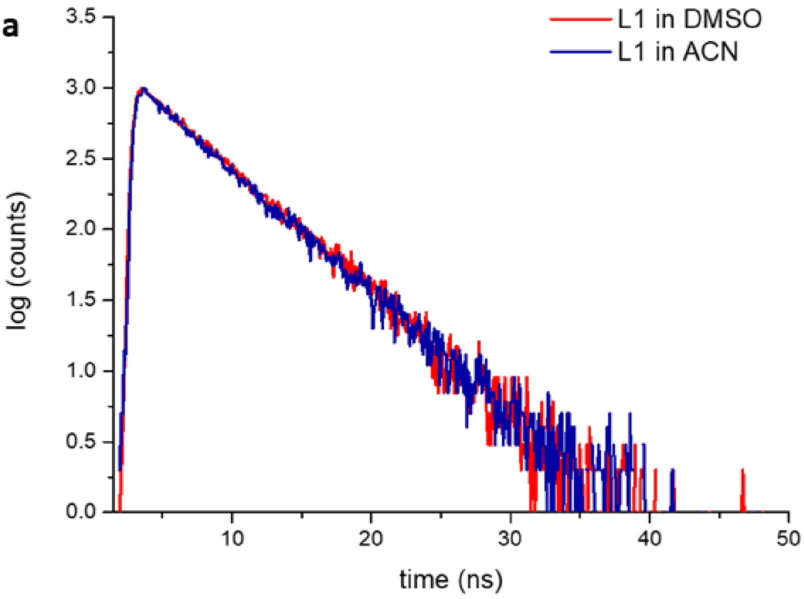

	DMSO	ACN
**τ (ns)**	4.9	4.9
χ^2	1.001	1.001

b

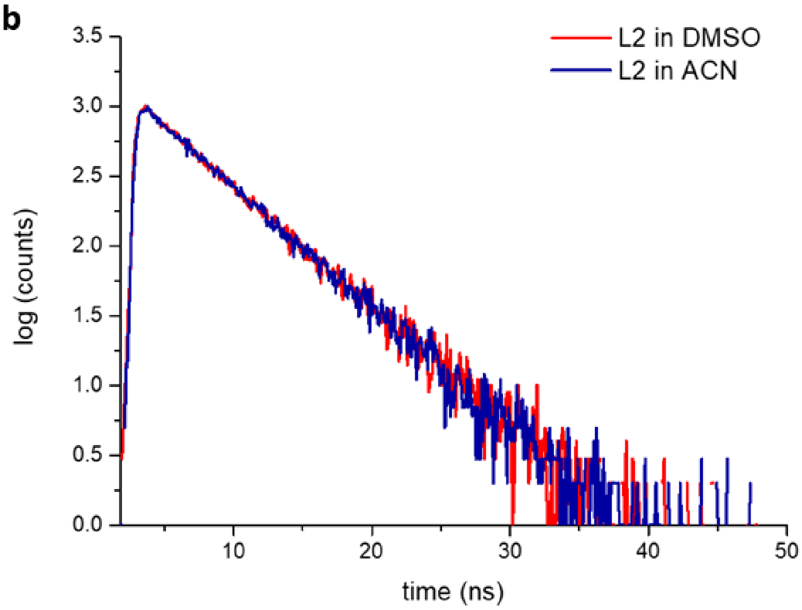

	DMSO	ACN
**τ (ns)**	4.9	4.9
χ^2	1.005	1.000

Figure S2. Excited state decay of a) **L1** and b) **L2** in ACN and DMSO (λexc = 400 nm).

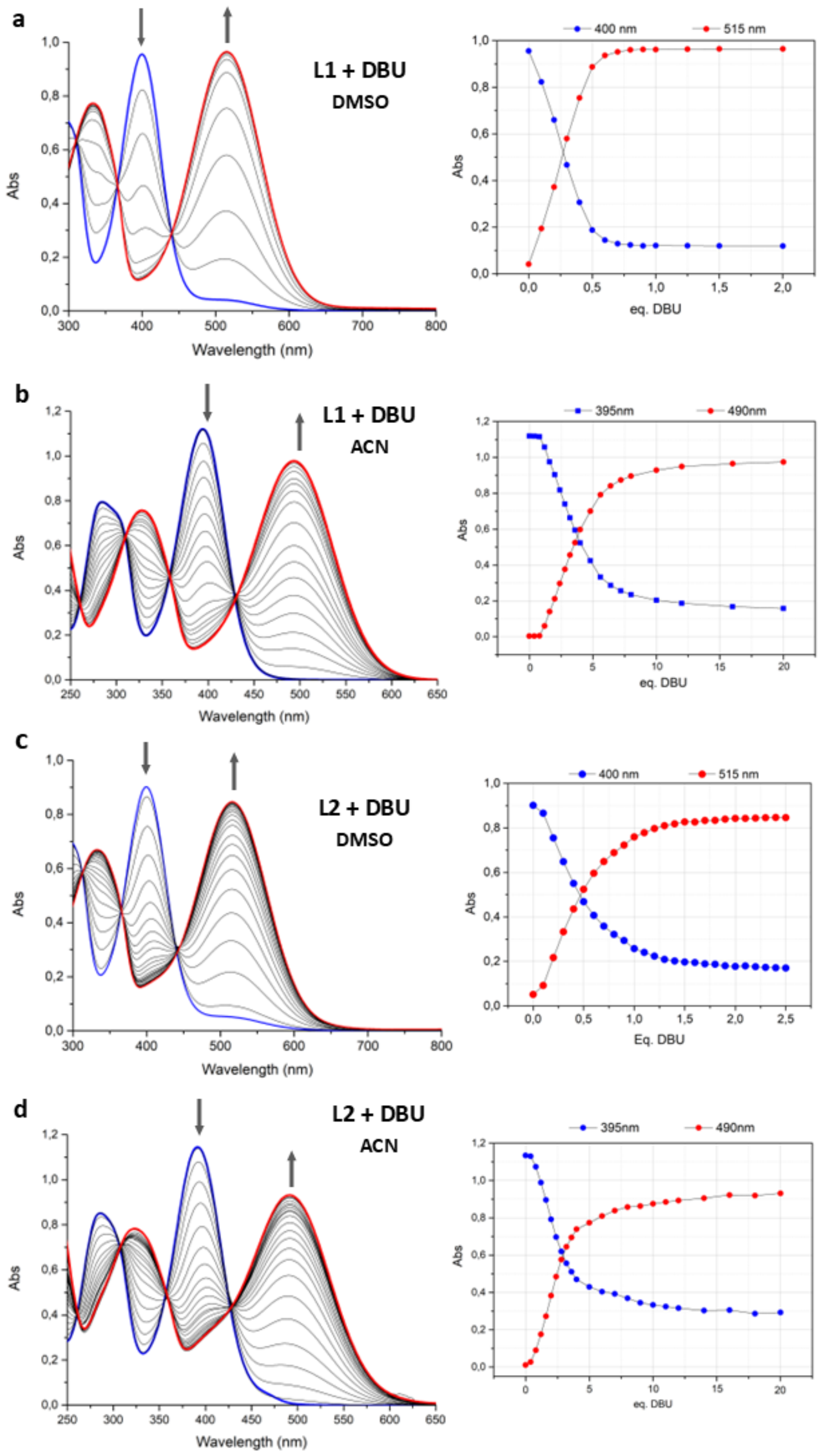

Figure S3. UV-Vis titrations of a) **L1** ($4\cdot10^{-5}$ mol · dm^{-3}) with DBU in DMSO (left) and trend of absorption intensity at 400 nm and 515 nm by increasing the eq. of base added (right); b) **L1** ($4\cdot10^{-5}$ mol · dm^{-3}) with DBU in ACN (left) and trend of absorption intensity at 395 nm and 490 nm by increasing the eq. of base added (right); c) **L2** ($2\cdot10^{-5}$ mol · dm^{-3}) with DBU in DMSO (left) and trend of absorption intensity at 400 nm and 515 nm by increasing the eq. of base added (right); d) **L2** ($2\cdot10^{-5}$ mol · dm^{-3}) with DBU in ACN (left) and trend of absorption intensity at 395 nm and 490 nm by increasing the eq. of base added (right). In all the experiments blue lines refer to the spectrum of the free Ligands and red lines to the spectrum registered after the last addition of base.

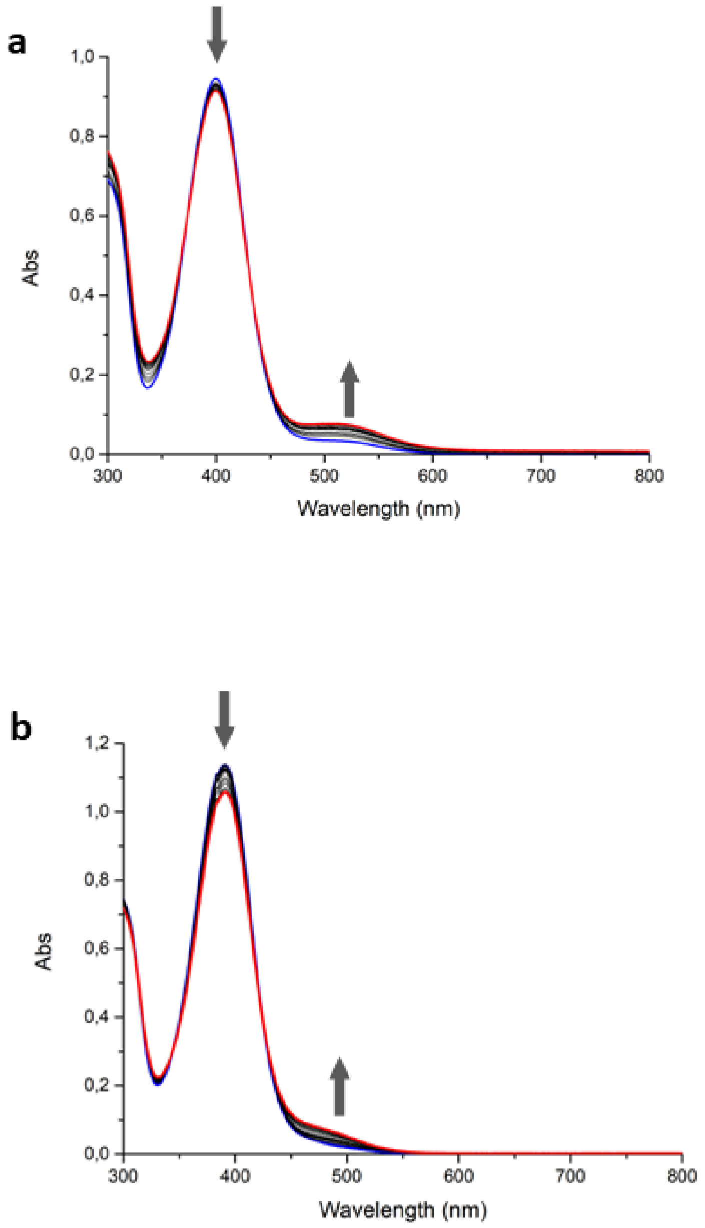

Figure S4. UV-Vis titrations of a) **L1** ($4 \cdot 10^{-5}$ mol · dm^{-3}) with DIPEA in DMSO; b) **L1** ($4 \cdot 10^{-5}$ mol · dm^{-3}) with DIPEA in ACN. In both experiments blue lines refer to the spectrum of the free Ligand and red lines to the spectrum registered after the last addition of base.

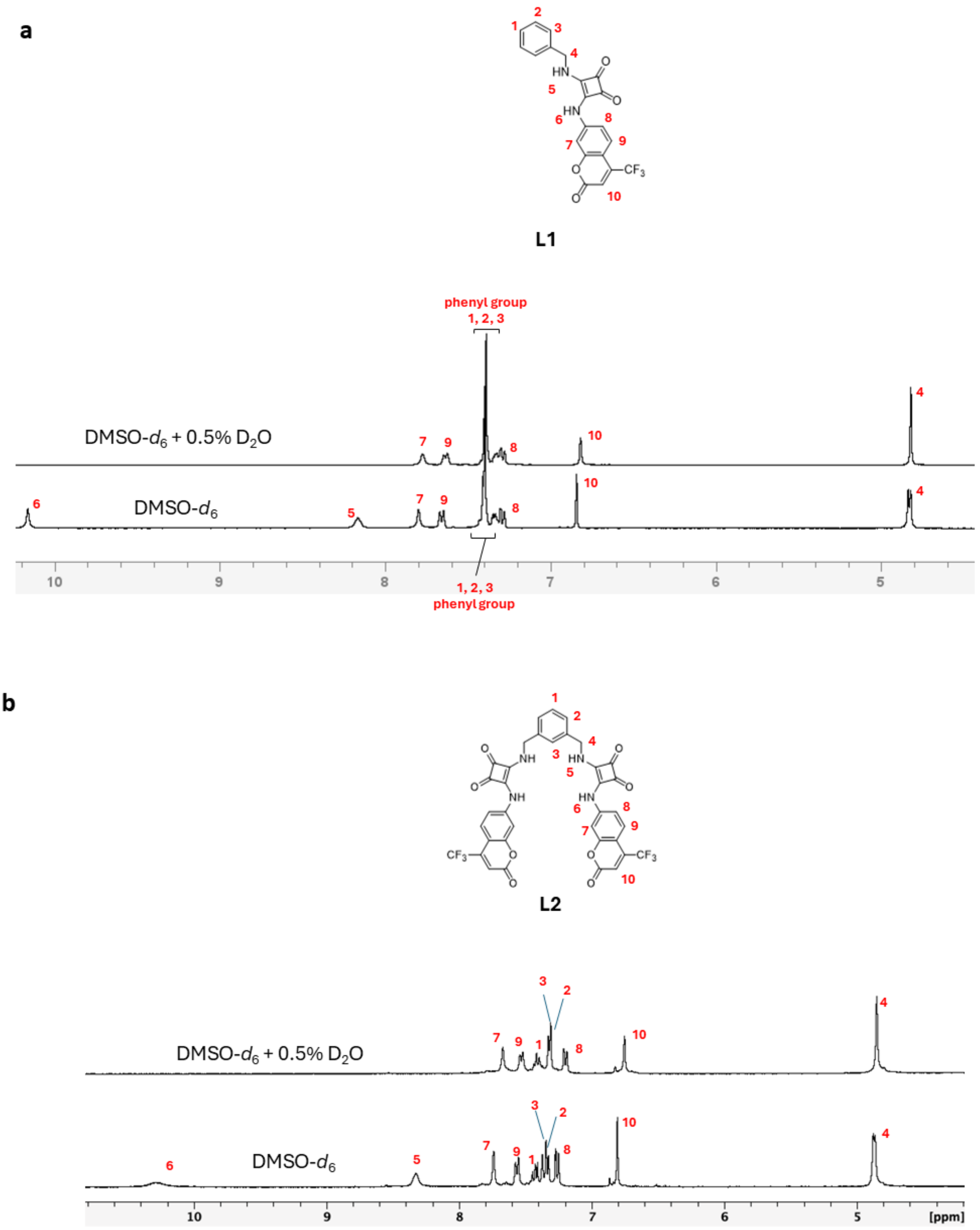

Figure S5. a) Stacked ^{1}H NMR spectra of **L1** registered in DMSO-d_6 and in DMSO-d_6 + 0.5% of D_2O; b) Stacked ^{1}H NMR spectra of **L2** registered in DMSO-d_6 and in DMSO-d_6 + 0.5% of D_2O. [**L1**] = [**L2**] = $7.5 \cdot 10^{-3}$ mol · dm^{-3}.

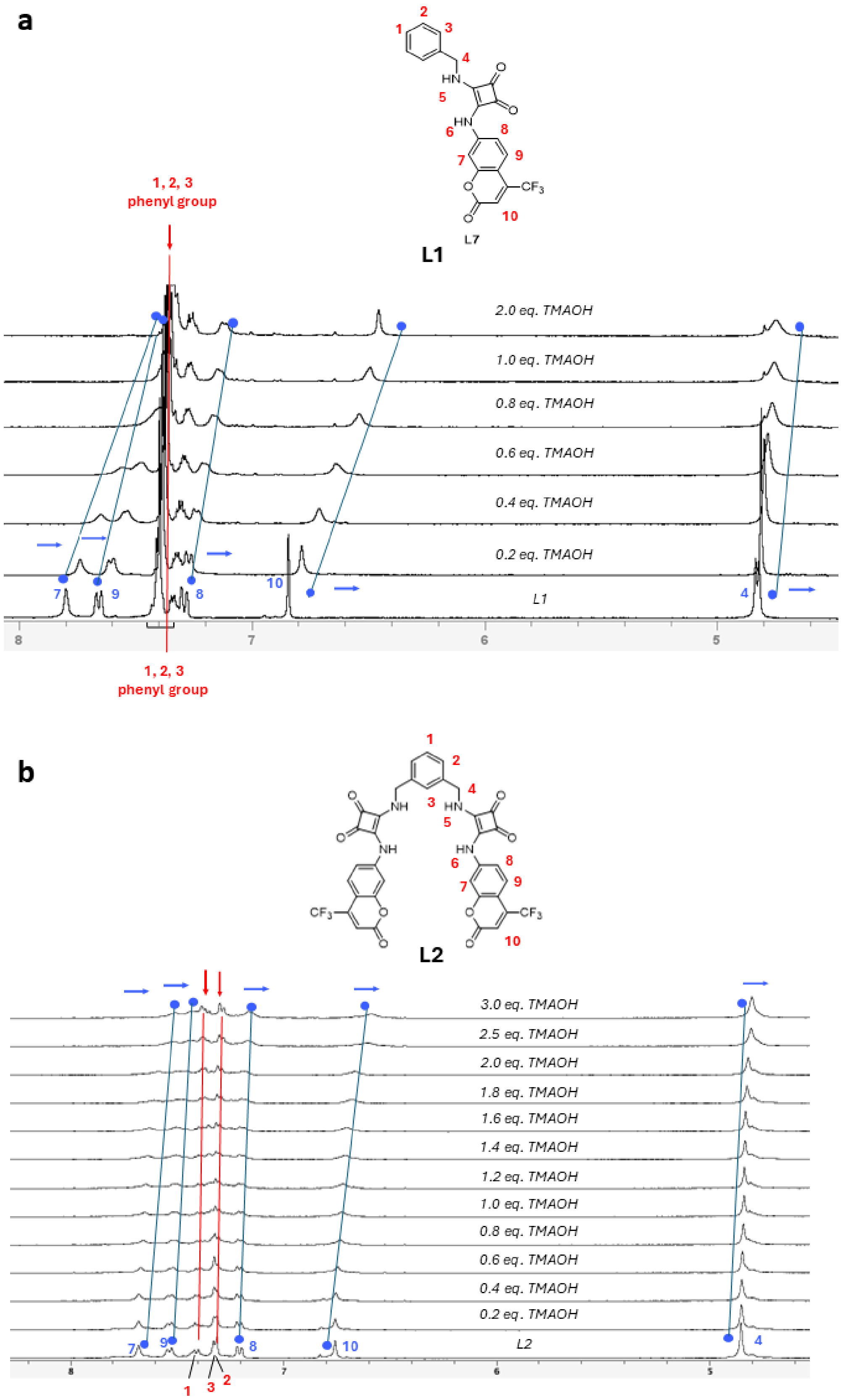

Figure S6. a) ^{1}H NMR spectra of **L1** registered in DMSO-d_6 + 0.5% of D_2O upon gradual addition of TMAOH. b) ^{1}H NMR spectra of **L2** registered in DMSO-d_6 + 0.5% of D_2O upon gradual addition of TMAOH. [**L1**] = [**L2**] = $7.5 \cdot 10^{-3}$ mol · dm^{-3}.

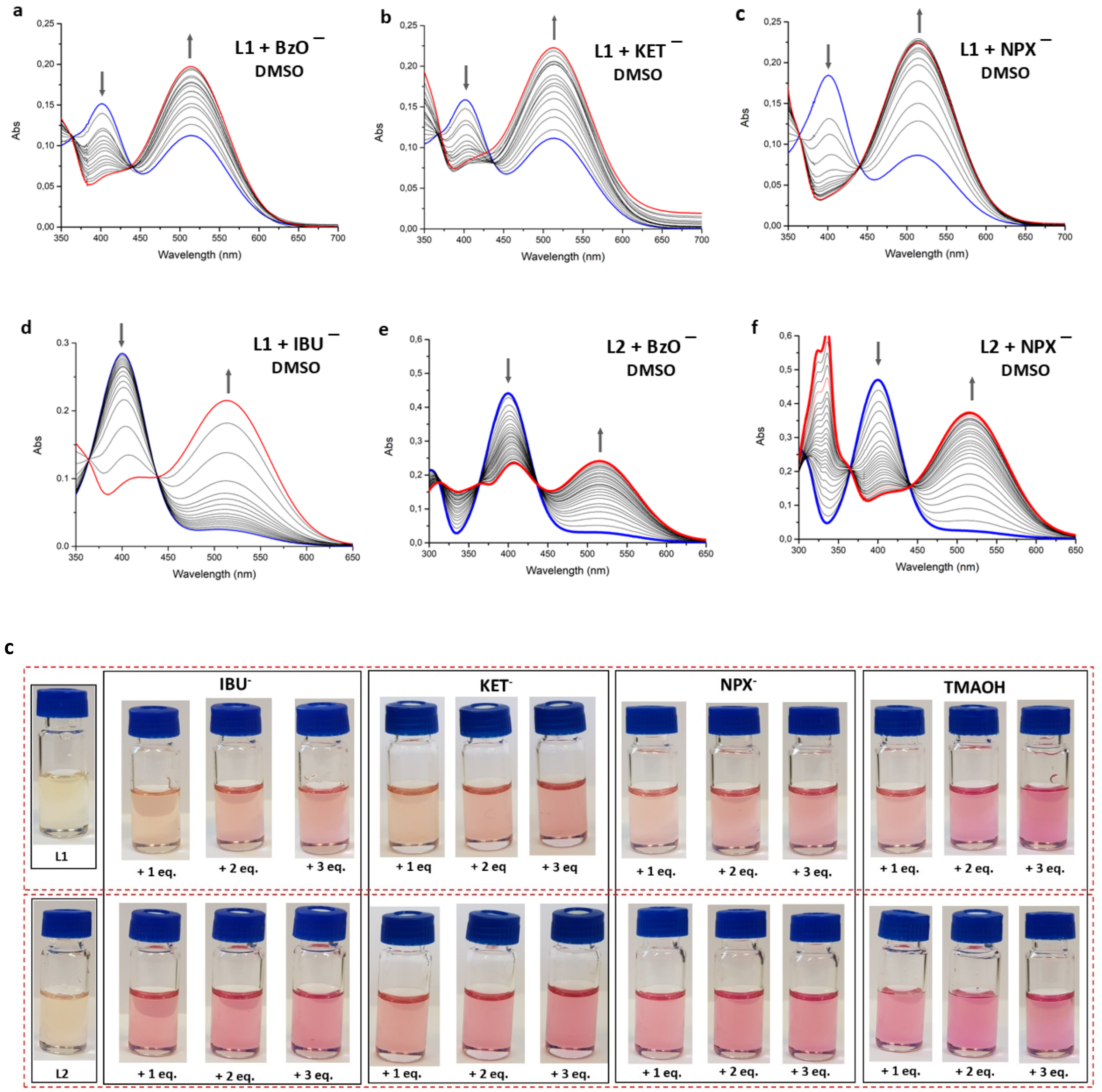

Figure S7. UV-Vis titrations performed in DMSO of **L1** upon gradually addition (0-10 eq.) of a) BzO^-, b) KET^-, c) NPX^-, d) IBU^-; and of **L2** in DMSO upon gradual addition (0-10 eq) of e) BzO^-, f) NPX^-. [**L1**] = [**L2**] = $1\cdot10^{-5}$ mol $\cdot$ dm^{-3}. Blue lines refer to the spectrum of the free ligands and red lines to the spectrum registered after the last addition of the anion; c) colour change after the addition of 1, 2 or 3 eq. of NSAIDs and TMAOH to a $1\cdot10^{-5}$ mol $\cdot$ dm^{-3} DMSO solution of **L1** and **L2**.

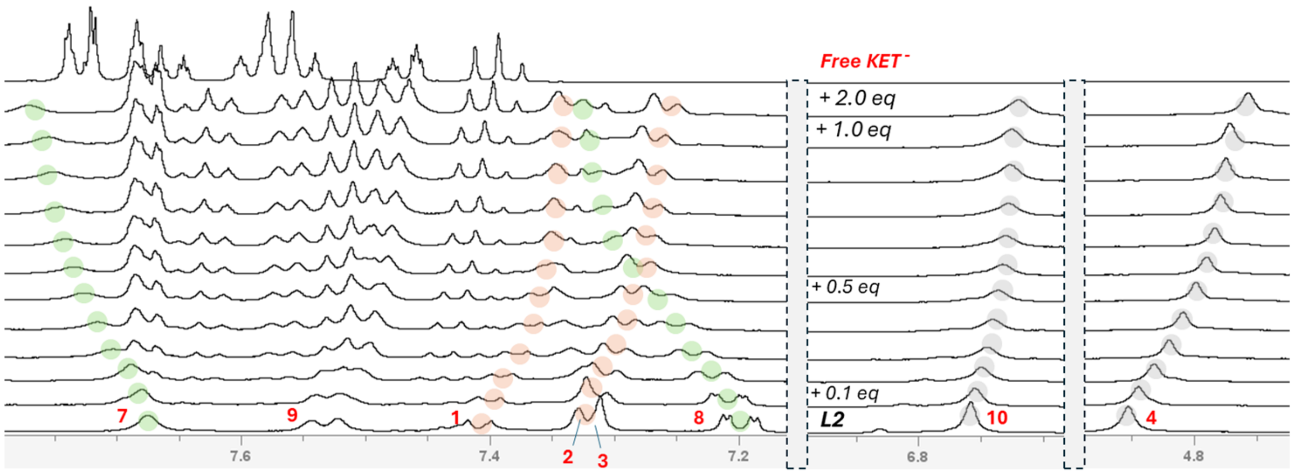

Figure S8. ^{1}H NMR spectra of **L2** registered in DMSO-d_6 + 0.5% of water upon gradual addition of sodium ketoprofen. [**L2**] = $7.5 \cdot 10^{-3}$ mol · dm^{-3}. The upper spectrum shows the signal of free sodium ketoprofen registered in DMSO-d_6 + 0.5% of water.

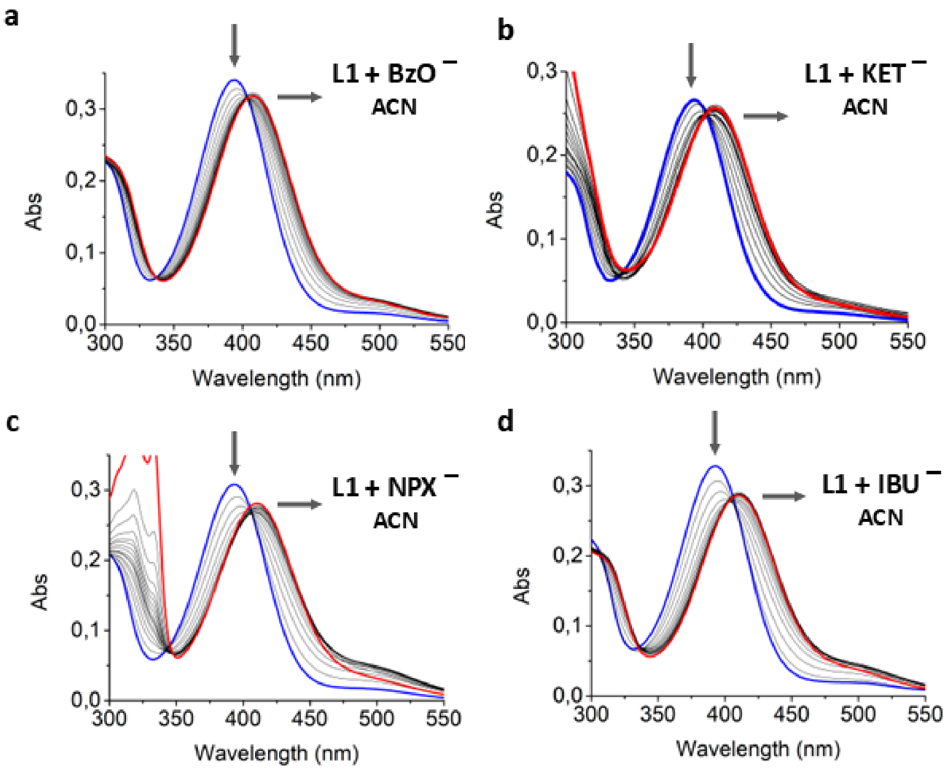

Figure S9. UV-Vis titrations performed in ACN of **L1** with gradual addition (0-10 eq.) of a) BzO^-, b) KET^-, c) NPX^-, d) IBU^- [**L1**] = $1 \cdot 10^{-5}$ mol · dm^{-3}. In all the experiments blue lines refer to the spectrum of the free Ligands and red lines to the spectrum registered after the last addition of the anion.

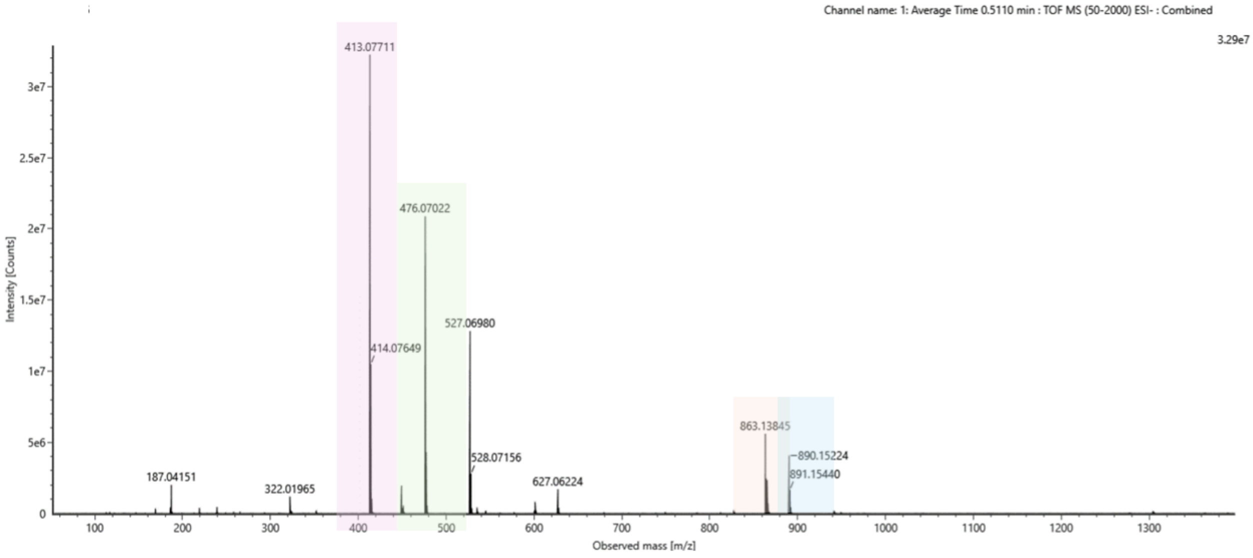

Figure S10. MS of **L1** in ACN (negative direct injection), z=1: 413 [**L1**-H]$^-$, 476 [**L1**-NO_3]$^-$, 863 [**L1$_2$**-Cl]$^-$, 890 [**L1$_2$**-NO_3]$^-$.

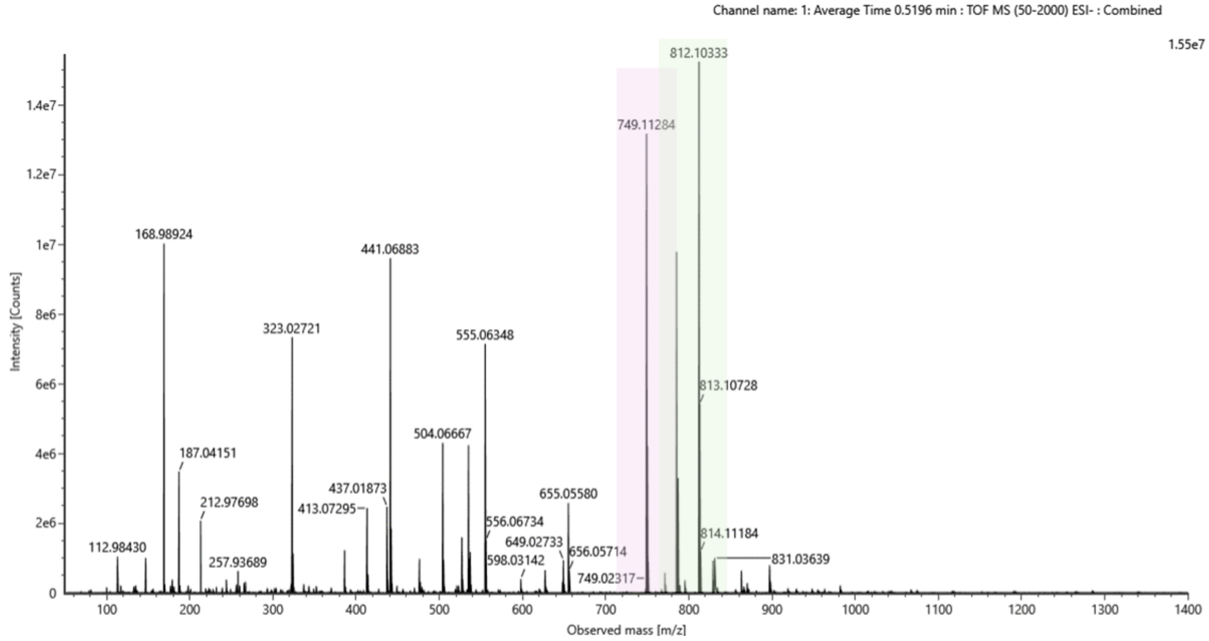

Figure S11. MS of **L2** in ACN (negative direct injection), z=1: 749 [**L2**-H]$^-$, 812 [**L2**-NO_3]$^-$.

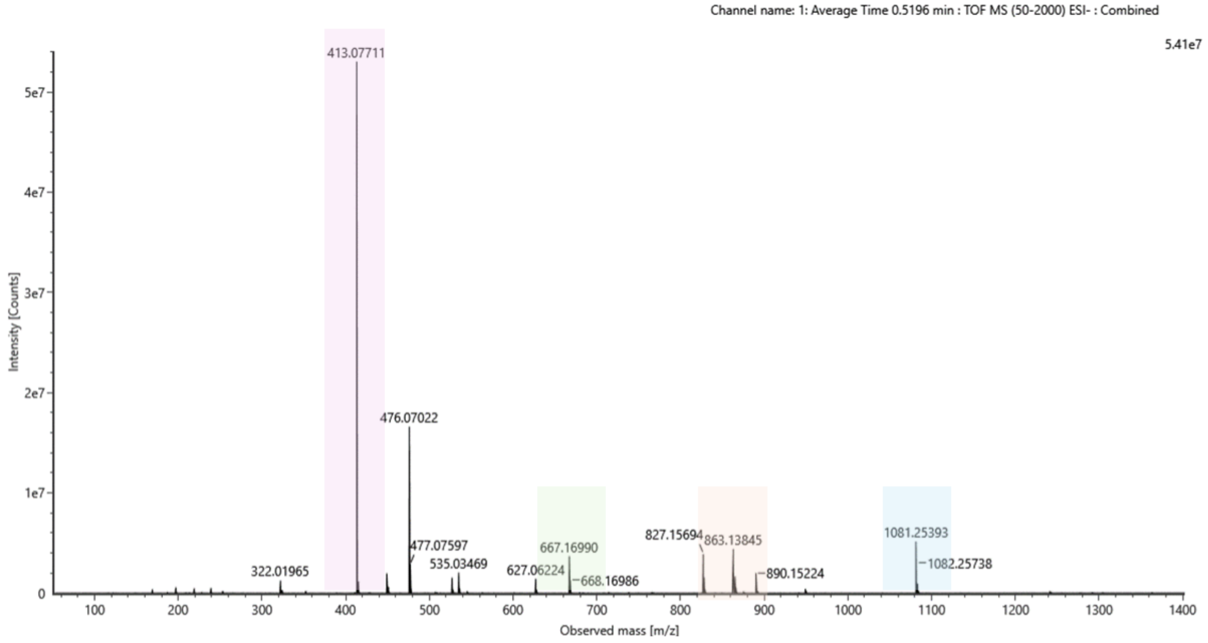

Figure S12. MS of **L1** *+ sodium ketoprofen* in ACN (negative direct injection), z=1: 413 [**L1**-H]$^-$, 667 [**L1**-KET]$^-$, 863 [**L1**$_2$-Cl]$^-$, 1081 [**L1**$_2$-KET]$^-$.

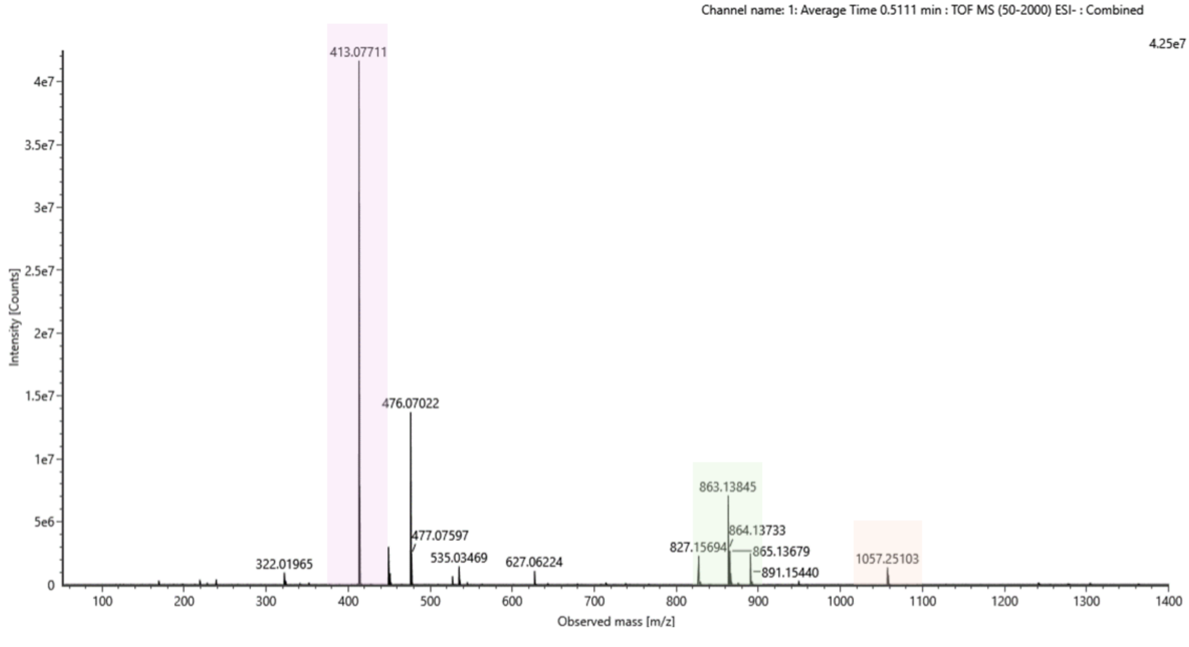

Figure S13. MS of **L1** *+ sodium naproxen* in ACN (negative direct injection), z=1: 413 [-H]$^-$, 863 [**L1**$_2$-Cl]$^-$, 1057 [**L1**$_2$-NPX]$^-$.

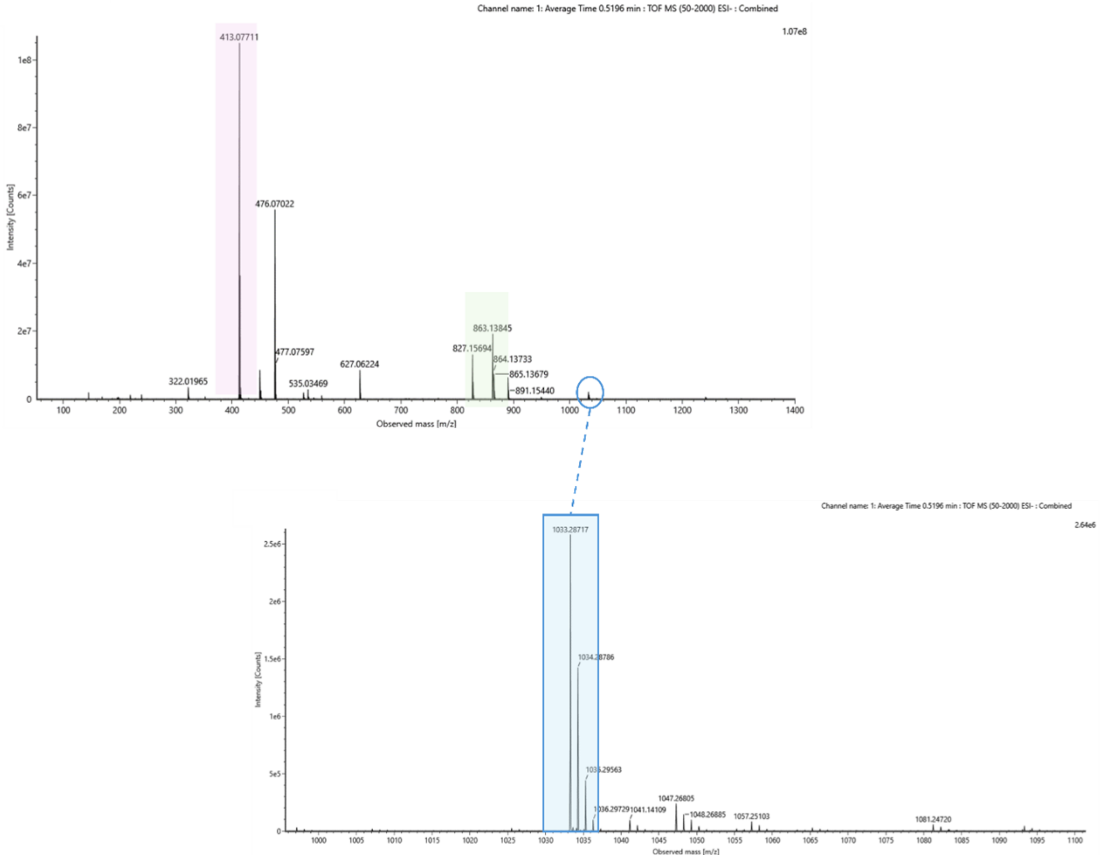

Figure S14. MS of **L1** + *sodium ibuprofen* in ACN (negative direct injection), z=1: 413 [**L1**-H]$^-$, 863 [$\mathbf{L1_2}$-Cl]$^-$, (zoom of) 1033 [$\mathbf{L1_2}$-IBU]$^-$.

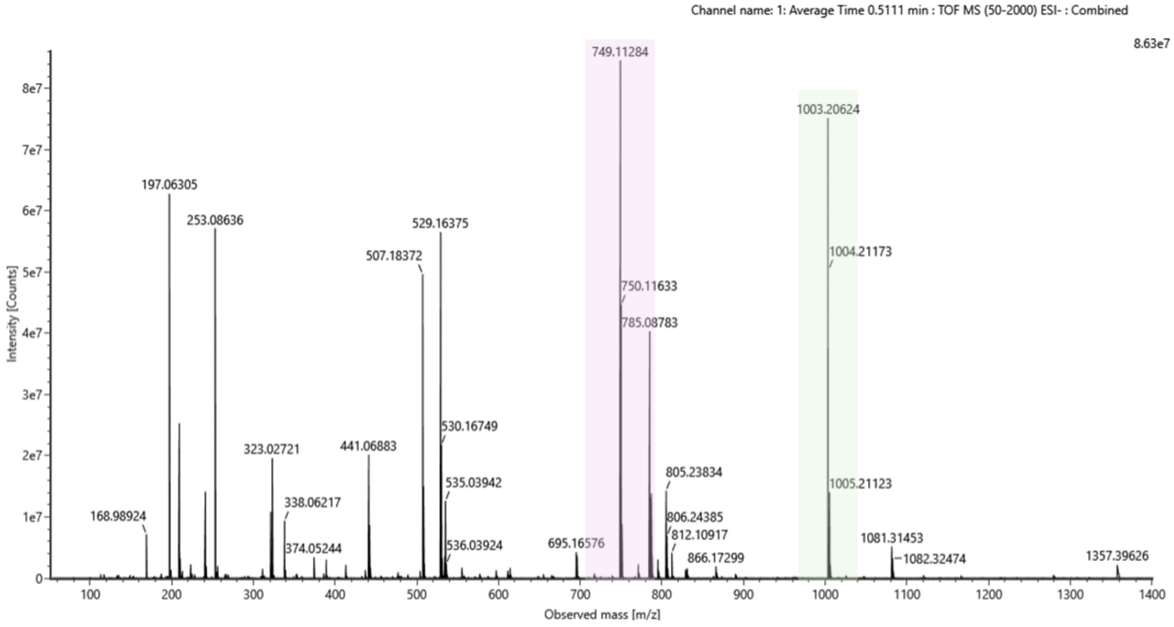

Figure S15. MS of **L2** + *sodium ketoprofen* in ACN (negative direct injection), z=1: 749 [**L2**-H]$^-$, 1033 [**L2**-KET]$^-$.

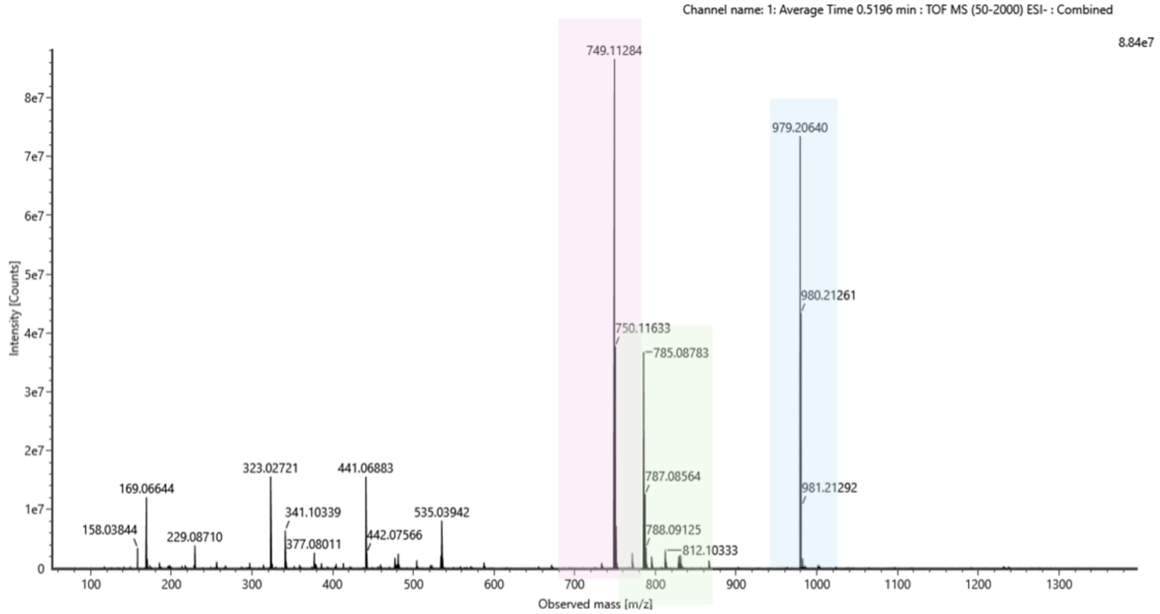

Figure S16. MS of **L2** + *sodium naproxen* in ACN (negative direct injection), z=1: 749 [**L2**-H]$^-$, 785 [**L2**-Cl]$^-$, 979 [**L2**-NPX]$^-$.

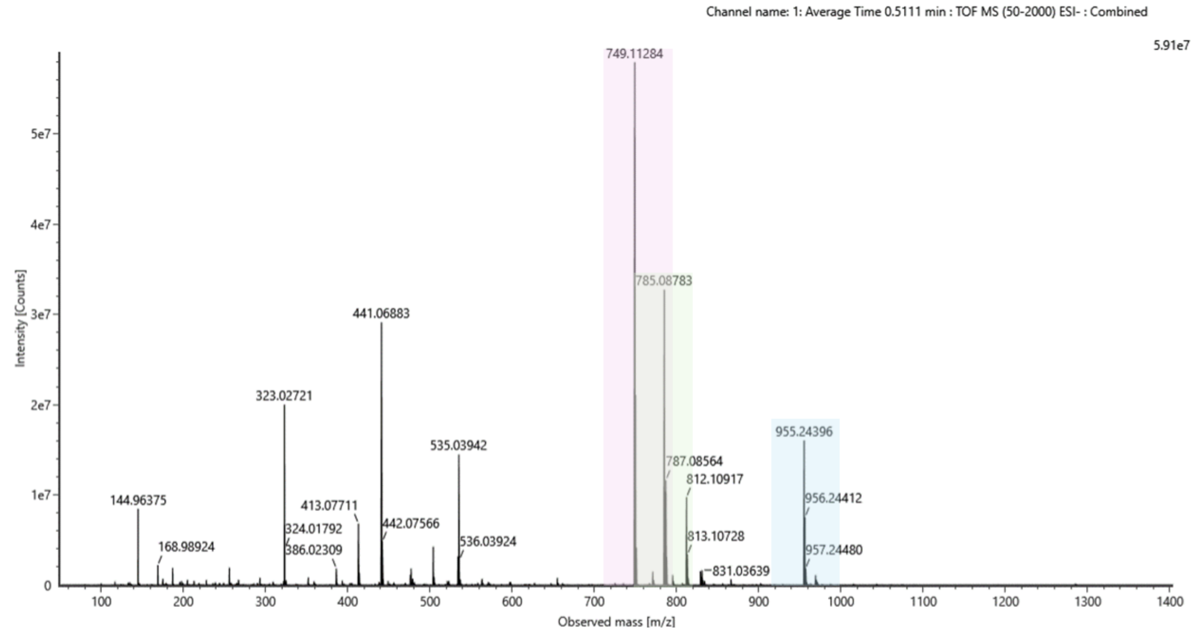

Figure S17. MS of **L2** + *sodium ibuprofen* in ACN (negative direct injection), z=1: 749 [**L2**-H]$^-$, 785 [**L2**-Cl]$^-$, 955 [**L2**-IBU]$^-$.

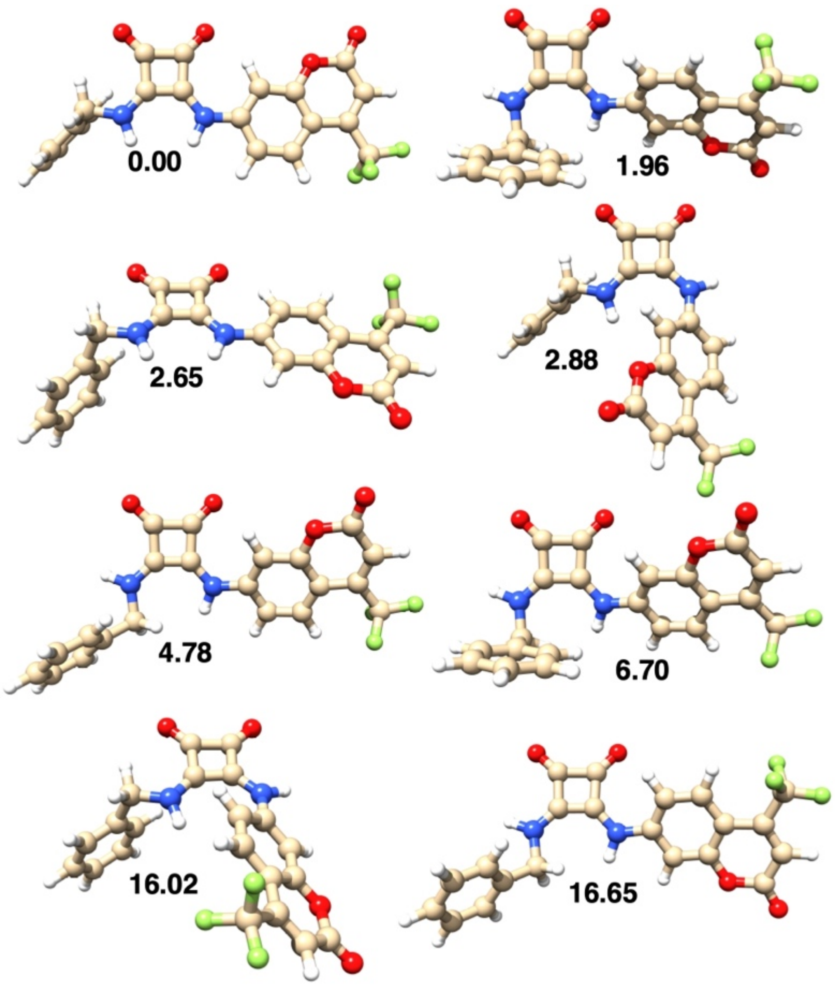

Figure S18. Structure of the conformers of **L1** obtained by DFT calculations in ACN and listed in order of increasing relative values of standard Gibbs free energy (in kJ/mol).

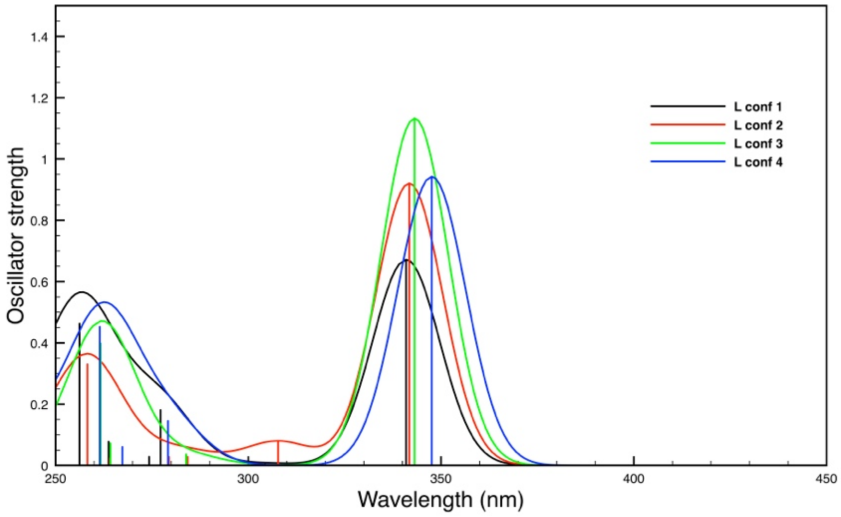

Figure S19. UV absorption spectra obtained by TD-DFT calculations in DMSO of the first four more stable conformations found for **L1** (see Figure 10).

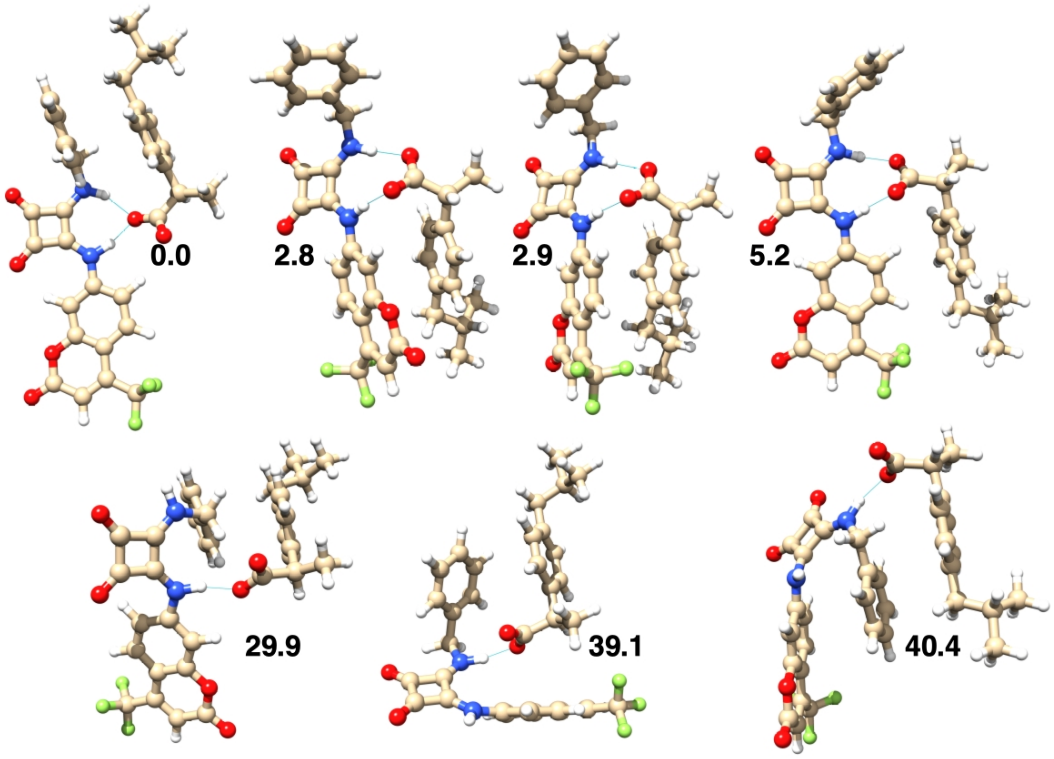

Figure S20. Structures of the isomers of the complexes between **L1** and IBU^{-}, obtained by DFT calculations in ACN and listed in order of increasing relative values of standard Gibbs free energy (in kJ/mol).

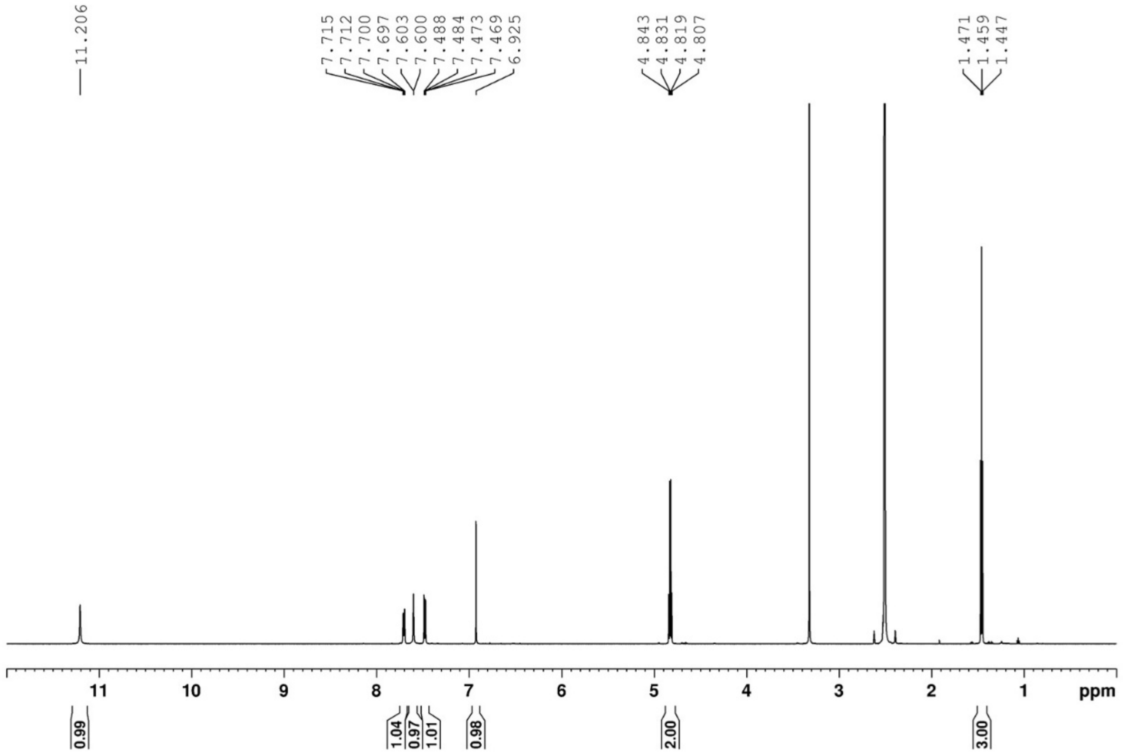

Figure S21. ^{1}H NMR spectrum of **3** in in DMSO-d_6.

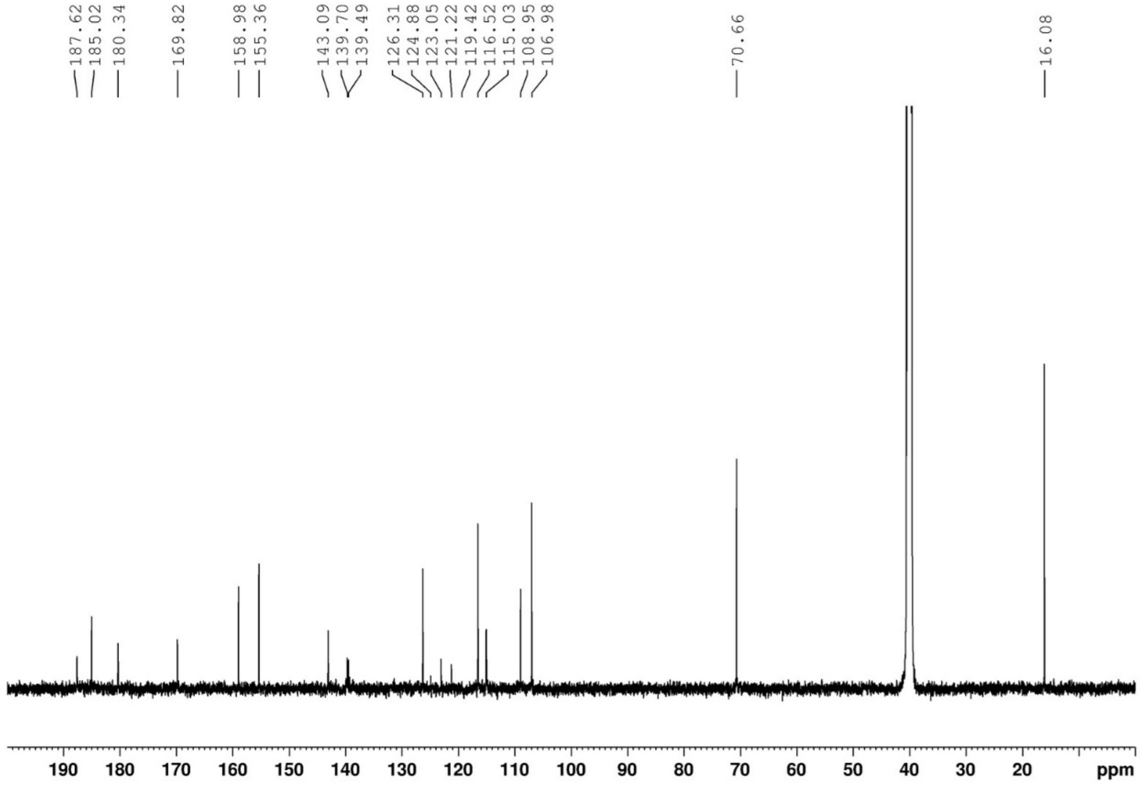

Figure S22. ^{13}C NMR spectrum of **3** in in DMSO-d_6.

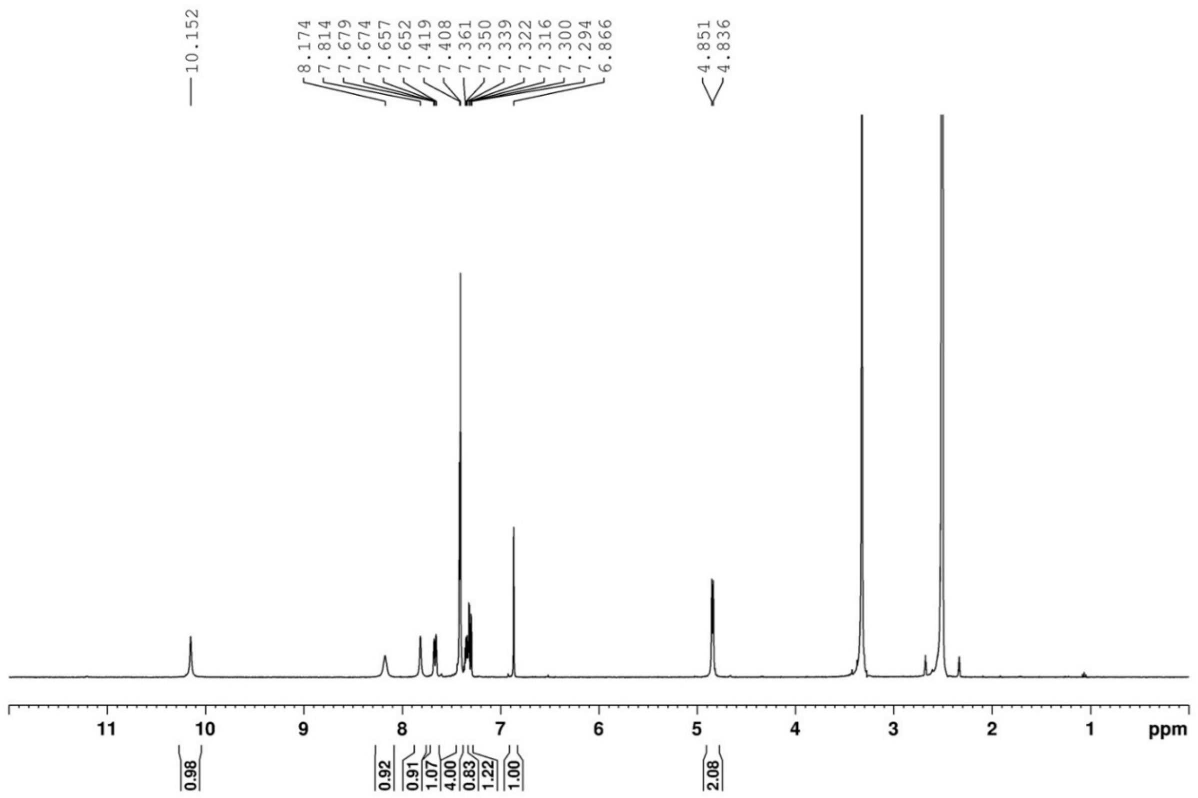

Figure S23. ^{1}H NMR spectrum of **L1** in in DMSO-d_6.

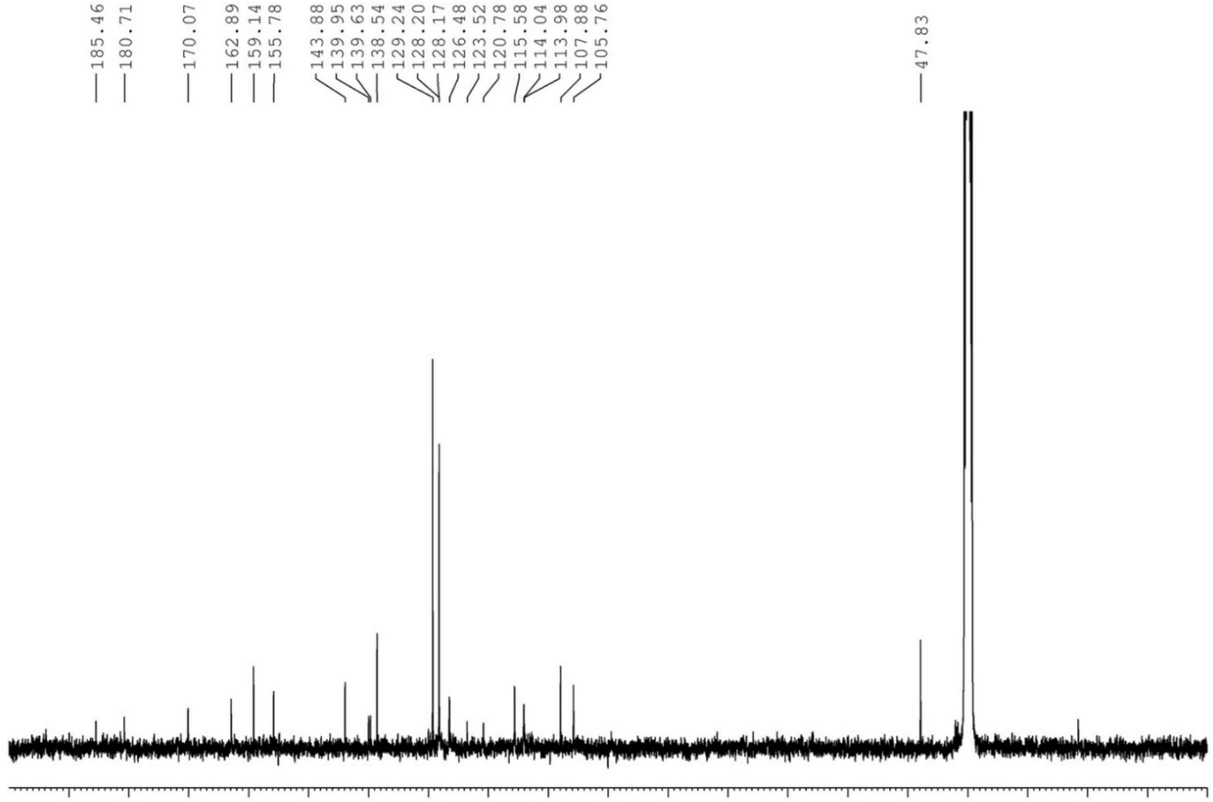

Figure S24. ^{13}C NMR spectrum of **L1** in in DMSO-d_6.

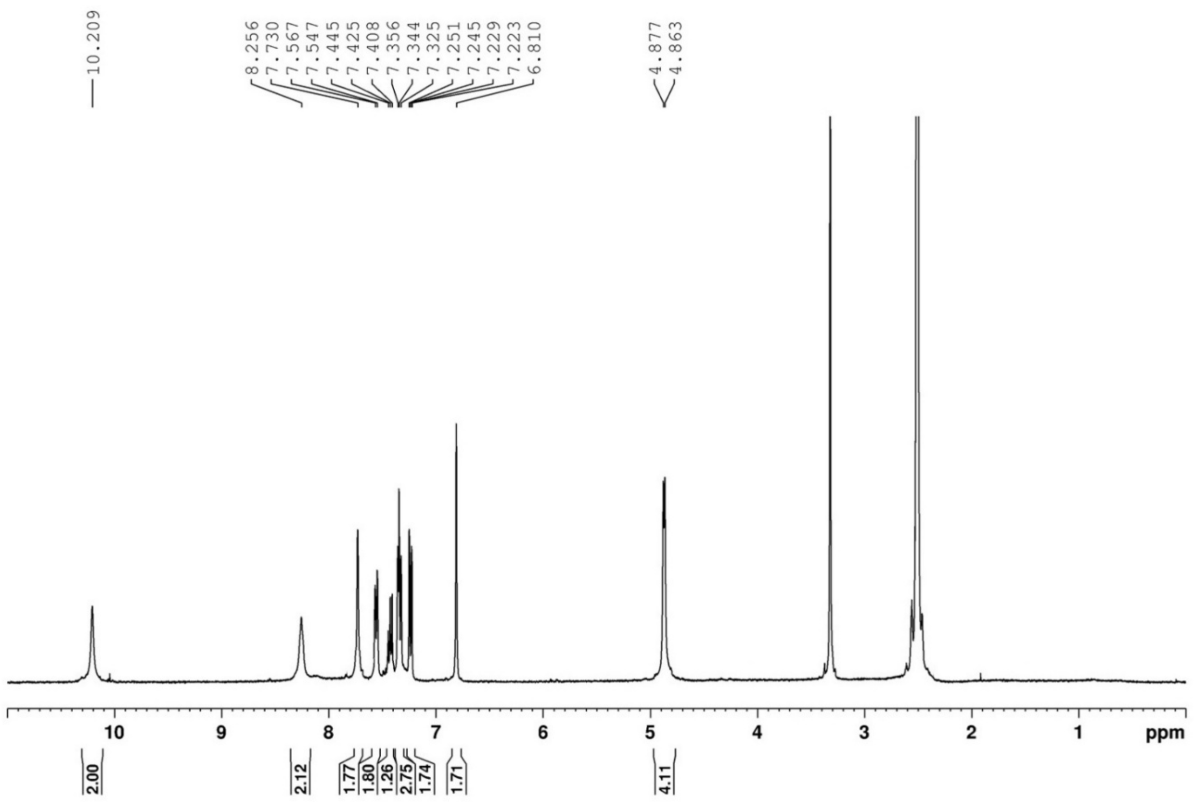

Figure S25. ^{1}H NMR spectrum of **L2** in in DMSO-*d*$_6$.

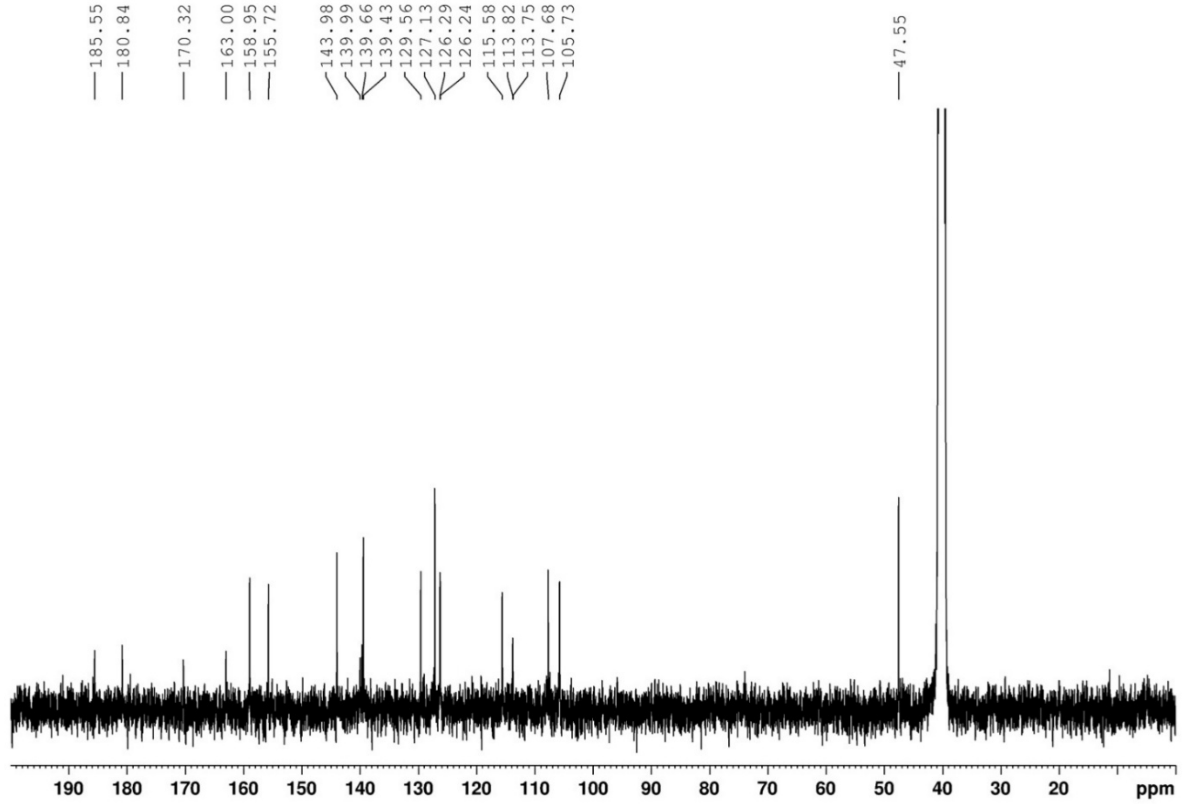

Figure S26. ^{13}C NMR spectrum of **L2** in in DMSO-*d*$_6$.
